# Annual Report to the Nation on the Status of Cancer, Part 1: National Cancer Statistics

**DOI:** 10.1093/jnci/djab131

**Published:** 2021-07-08

**Authors:** Farhad Islami, Elizabeth M Ward, Hyuna Sung, Kathleen A Cronin, Florence K L Tangka, Recinda L Sherman, Jingxuan Zhao, Robert N Anderson, S Jane Henley, K Robin Yabroff, Ahmedin Jemal, Vicki B Benard

**Affiliations:** 1 Department of Surveillance and Health Equity Science, American Cancer Society, Atlanta, GA, USA; 2 North American Association of Central Cancer Registries, Springfield, IL, USA; 3 Division of Cancer Control and Population Sciences, National Cancer Institute, National Institutes of Health, Bethesda, MD, USA; 4 Division of Cancer Prevention and Control, National Center for Chronic Disease Prevention and Health Promotion, Centers for Disease Control and Prevention, Atlanta, GA, USA; 5 National Center for Health Statistics, Centers for Disease Control and Prevention, Hyattsville, MD, USA

## Abstract

**Background:**

The American Cancer Society, Centers for Disease Control and Prevention, National Cancer Institute, and North American Association of Central Cancer Registries collaborate to provide annual updates on cancer incidence and mortality and trends by cancer type, sex, age group, and racial/ethnic group in the United States. In this report, we also examine trends in stage-specific survival for melanoma of the skin (melanoma).

**Methods:**

Incidence data for all cancers from 2001 through 2017 and survival data for melanoma cases diagnosed during 2001-2014 and followed-up through 2016 were obtained from the Centers for Disease Control and Prevention- and National Cancer Institute-funded population-based cancer registry programs compiled by the North American Association of Central Cancer Registries. Data on cancer deaths from 2001 to 2018 were obtained from the National Center for Health Statistics’ National Vital Statistics System. Trends in age-standardized incidence and death rates and 2-year relative survival were estimated by joinpoint analysis, and trends in incidence and mortality were expressed as average annual percent change (AAPC) during the most recent 5 years (2013-2017 for incidence and 2014-2018 for mortality).

**Results:**

Overall cancer incidence rates (per 100 000 population) for all ages during 2013-2017 were 487.4 among males and 422.4 among females. During this period, incidence rates remained stable among males but slightly increased in females (AAPC = 0.2%, 95% confidence interval [CI] = 0.1% to 0.2%). Overall cancer death rates (per 100 000 population) during 2014-2018 were 185.5 among males and 133.5 among females. During this period, overall death rates decreased in both males (AAPC = −2.2%, 95% CI = −2.5% to −1.9%) and females (AAPC = −1.7%, 95% CI = −2.1% to −1.4%); death rates decreased for 11 of the 19 most common cancers among males and for 14 of the 20 most common cancers among females, but increased for 5 cancers in each sex. During 2014-2018, the declines in death rates accelerated for lung cancer and melanoma, slowed down for colorectal and female breast cancers, and leveled off for prostate cancer. Among children younger than age 15 years and adolescents and young adults aged 15-39 years, cancer death rates continued to decrease in contrast to the increasing incidence rates. Two-year relative survival for distant-stage skin melanoma was stable for those diagnosed during 2001-2009 but increased by 3.1% (95% CI = 2.8% to 3.5%) per year for those diagnosed during 2009-2014, with comparable trends among males and females.

**Conclusions:**

Cancer death rates in the United States continue to decline overall and for many cancer types, with the decline accelerated for lung cancer and melanoma. For several other major cancers, however, death rates continue to increase or previous declines in rates have slowed or ceased. Moreover, overall incidence rates continue to increase among females, children, and adolescents and young adults. These findings inform efforts related to prevention, early detection, and treatment and for broad and equitable implementation of effective interventions, especially among under resourced populations.

The Centers for Disease Control and Prevention (CDC), the American Cancer Society, the National Cancer Institute (NCI), and the North American Association of Central Cancer Registries (NAACCR) have collaborated annually since 1998 to provide updated information about cancer occurrence and trends by cancer type, sex, age group, and racial/ethnic group in the United States. Part 1 of this report focuses on national cancer statistics and highlights trends in stage-specific survival for melanoma of the skin, the first cancer for which effective immune checkpoint inhibitors were developed (1). Part 2 focuses on the economic burden of cancer in the United States (2).

## Methods

### Data Sources

####  

##### Cancer Incidence Data

Population-based cancer incidence data by age, sex, and race and ethnicity were obtained from registries that participate in the CDC’s National Program of Cancer Registries and/or the NCI’s Surveillance, Epidemiology, and End Results (SEER) Program. Only registries whose data satisfied NAACCR’s criteria for data quality and completeness were included in this study (3). For rate analyses, 49 states and 1 territory (Puerto Rico) met data criteria for every year during 2013-2017, and for trend analyses, 46 states met data criteria for every year during 2001-2017, representing 99% and 92% of the population of the United States and Puerto Rico, respectively. States included in any analysis in this report are listed in corresponding figure legends and table footnotes.

Anatomic site and histology were coded according to International Classification of Diseases (ICD) for Oncology 3rd edition (4) and categorized according to SEER site groups (5). Only cases of cancer defined as malignant were included in this report, except that in situ and malignant bladder cancers were combined when reporting bladder cancer incidence rates.

##### Cancer Mortality Data

Cause of death by age, sex, race, and ethnicity (2001-2018) from all 50 states and the District of Columbia was based on death certificate information reported to state vital statistics offices and compiled through CDC’s National Center for Health Statistics’ (NCHS) National Vital Statistics System (6). The underlying causes of death were selected according to ICD-10, then categorized according to SEER site groups to maximize comparability with ICD-O classifications (5).

##### Survival Data for Melanoma of the Skin

Survival data for cases of malignant melanoma of the skin (melanoma) diagnosed from 2001 through 2014 were from data compiled by NAACCR from registries in 28 states—covering 86% of the US population—considered to have sufficient vital status follow-up to conduct survival analyses during the entire study period (7) meeting the NAACCR standard, that is, death ascertainment through the study cutoff date of December 31, 2016, or follow-up dates on or after January 1, 2017, for a minimum of 90% of patients (8). Cases were censored at an achieved age of 100 years. Cases identified by death certificate or autopsy only or without survival data were excluded from the survival analysis.

##### Population Data

Population estimates by age, sex, race, and Hispanic origin were a modification of intercensal (for July 1, 2001-2009) and Vintage 2017 (for July 1, 2010-2018) annual county population estimates produced by the US Census Bureau’s Population Estimates Program in collaboration with NCHS and with support from the NCI (9). The estimates incorporate bridged single-race estimates derived from the original multiple-race categories in the 2000 and 2010 censuses (10).

##### Demographic Characteristics

Rates and trends are presented by sex, racial/ethnic group, and age (all ages, children aged 0-14 years, and adolescents and young adults [AYA] aged 15-39 years). Information about race and ethnicity was collected separately and was based on information abstracted from medical records for incidence in cancer registries or death certificates from NCHS for mortality. In this report, information about race and ethnicity was combined to create 5 mutually exclusive racial and ethnic (racial/ethnic) groups: non-Hispanic White (White), non-Hispanic Black (Black), non-Hispanic American Indian or Alaska Native (AI/AN), non-Hispanic Asian or Pacific Islander (API), and Hispanic (any race). Race information for AI/AN was considered reliable only for geographic areas covered by the Indian Health Service Purchased/Referred Care Delivery Areas; thus, to minimize racial misclassification for AI/AN, incidence and mortality data for this group were based only on counties covered by Indian Health Service Purchased/Referred Care Delivery Areas in states that provided county-level information (11). Persons with other or unknown race or unknown ethnicity were included in overall rates but were not included as separate categories.

### Statistical Analysis

Cross-sectional incidence (2013-2017) and death (2014-2018) rates for all ages combined, children, and AYA by cancer type and racial/ethnic group were calculated using SEER*Stat software, version 8.3.6 (12). All rates were age-standardized to the 2000 US standard population and were expressed per 100 000 standard population. Corresponding 95% confidence intervals (CIs) were calculated as modified gamma intervals and allow for informal comparisons between groups, without specifying a referent group. Rates based on fewer than 16 cases or deaths during the 5-year period were deemed to be statistically unreliable and were suppressed. Because delays in reporting of cancer cases to cancer registries can cause incidence rates to be underestimated, all case counts and incidence rates were adjusted for reporting delay (13). Incidence and death rates and trends are reported for males and females for each cancer type that ranked in the top 15 incident cancers or causes of cancer death for any racial/ethnic group. For children, incidence and death rates and trends are presented for the 3 and 2 most common cancer types, respectively; these numbers for AYA were 6 and 4, respectively.

Temporal trends in delay- and age-adjusted incidence (2001-2017) and age-adjusted death (2001-2018) rates were estimated using joinpoint regression (14). A maximum number of 3 joinpoints (4 line segments) were allowed for both incidence and deaths. Annual percent change (APC) characterizes the slope of a single segment, and average APC (AAPC), a summary measure over a fixed interval. Two-sided statistically significant (*P *<* *.05) differences from zero were determined using a *t* test for the APC and for the AAPC when it laid entirely within the last joinpoint segment and a z-test when the last joinpoint fell within the last 5 years of data. When the slope of the trend (APC or AAPC) was statistically significant, the trend was considered increasing (slope >0) or decreasing (slope <0). Trends based on fewer than 10 cases or deaths in any of the data years were considered statistically unreliable and were suppressed. Corresponding 95% confidence intervals for trends were calculated using the parametric method and allow for informal conservative comparisons between groups.

Two-year age-standardized relative survival for melanoma cases diagnosed during 2001-2014 and followed-up through 2016 was calculated based on complete dates and monthly intervals using the Ederer II actuarial method (8,12). We chose 2-year relative survival to have multiple data points to examine trends in survival following the introduction of new therapies, first of which was approved by the Food and Drug Administration (FDA) in 2011 (15). Annual trends in 2-year age-standardized relative survival and 95% confidence intervals were estimated fitting a proportional hazard joinpoint model to survival data on the log hazard scale using the NCI’s JPSurv software (16), with a maximum of 2 joinpoints (3 line segments). All survival estimates were age-standardized using the International Cancer Survival Standards, age standard 2, and age groups 15-44 years, 45-54 years, 55-64 years, 65-74 years, and 75 years and older (8).

## Results

### Cancer Incidence Rates and Trends

**Figure 1.**
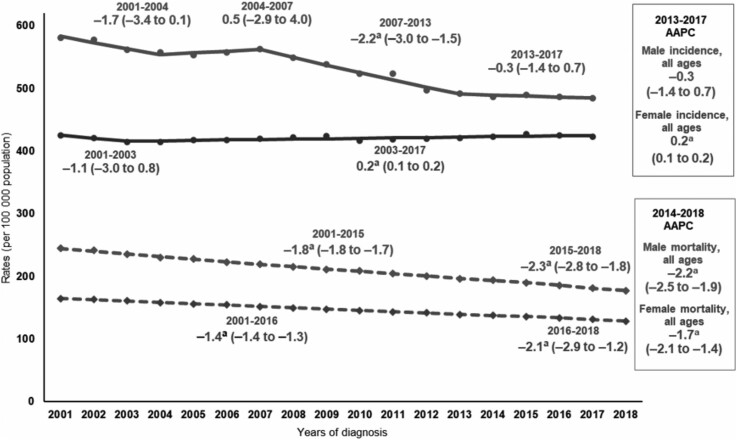
Trends in age -standardized incidence (2001-2017) and mortality (2001-2018) rates, all cancer sites combined, all ages, all races and ethnicities combined, by sex. Trends were estimated using joinpoint regression and characterized by the annual percent change (APC), the slope of a single segment, and the average APC (AAPC), a summary measure of the APCs over a fixed 5-year interval. Joinpoint models with up to 3 joinpoints are based on rates per 100 000 population and are age standardized to the 2000 US standard population (19 age groups, Census P25–1130). Incidence rates were delay adjusted and covered 92% of the US population, and mortality covered the entire United States. Registries included for incidence (46 states): Alabama, Alaska, Arizona, Arkansas, California, Colorado, Connecticut, Delaware, Florida, Georgia, Hawaii, Idaho, Illinois, Indiana, Iowa, Kansas, Kentucky, Louisiana, Maine, Maryland, Massachusetts, Michigan, Minnesota, Missouri, Montana, Nebraska, New Hampshire, New Jersey, New Mexico, New York, North Carolina, North Dakota, Ohio, Oklahoma, Oregon, Pennsylvania, Rhode Island, South Carolina, South Dakota, Texas, Utah, Vermont, Washington, West Virginia, Wisconsin, and Wyoming. Scattered points were observed rates; lines were fitted rates according to joinpoint regression. ^a^The APC or AAPC is statistically significantly different from 0 (2-sided *P *<* *.05), using a *t* test for the APC and for the AAPC when it laid entirely within the last joinpoint segment and a z-test when the last joinpoint fell within the last 5 years of data. The 95% confidence limits are given in parentheses.

The overall cancer incidence rate (per 100 000 population) in 2013-2017 was 448.3, with rates higher in males (487.4) than in females (422.4) (Table 1). During the most recent 5 years (2013-2017), incidence rates were stable in both sexes combined and among males but increased slightly among females (AAPC = 0.2%, 95% CI = 0.1% to 0.2%). Longer-term trends (2001-2017) in cancer incidence rates also varied by sex (Figure 1; Table 2); among men, rates were stable during 2001-2007, decreased an average of 2.2% per year during 2007-2013, and became stable again during 2013-2017. Among females, overall cancer incidence rates were stable during 2001-2003 and increased slightly during 2003-2017 (APC = 0.2%, 95% CI = 0.1% to 0.2%).

**Table 1.** Age-standardized, delay-adjusted incidence rates and fixed-interval trends (2013-2017) for the most common cancers, all ages, by sex, age group, and racial/ethnic group, for areas in the United States with high-quality incidence data^a^

Sex and cancer site or type^b^	Rank	All racial/ethnic groups combined^c^			Non-Hispanic White^c^^ ^			Non-Hispanic Black^c^			Non-Hispanic API^c^			Non-Hispanic AI/AN (PRCDA)^ c^			Hispanic^c^		
		Rate^d^ (95% CI)	AAPC^e^ (95% CI)	*P*	Rate^d^ (95% CI)	AAPC^e^ (95% CI)	*P*	Rate^d^ (95% CI)	AAPC^e^ (95% CI)	*P*	Rate^d^ (95% CI)	AAPC^e^ (95% CI)	*P*	Rate^d^ (95% CI)	AAPC^e^ (95% CI)	*P*	Rate^d^ (95% CI)	AAPC^e^ (95% CI)	*P*
All sites																			
Both sexes combined	—	448.3 (448.0 to 448.6)	0.0 (−0.7 to 0.7)	.98	468.3 (467.9 to 468.7)	0.2 (−0.5 to 0.9)	.45	463.4 (462.4 to 464.4)	−0.7 (−1.0 to −0.4)	.001	296.4 (295.2 to 297.5)	−0.3 (−0.4 to −0.2)	<.001	399.1 (394.6 to 403.7)	−0.2 (−0.4 to 0.0)	.06	348.7 (347.9 to 349.6)	−0.7 (−0.8 to −0.6)	<.001
Males	—	487.4 (486.9 to 487.8)	−0.3 (−1.4 to 0.7)	.48	503.0 (502.5 to 503.6)	−0.1 (−1.1 to 1.0)	.85	540.7 (539.0 to 542.4)	−1.1 (−2.1 to −0.1)	.03	298.5 (296.7 to 300.3)	−1.3 (−1.5 to −1.2)	<.001	419.1 (412.0 to 426.4)	−0.8 (−1.2 to −0.5)	<.001	375.3 (373.9 to 376.6)	−1.0 (−1.6 to −0.5)	.003
Females	—	422.4 (422.0 to 422.8)	0.2 (0.1 to 0.2)	.002	445.8 (445.3 to 446.3)	0.3 (0.2 to 0.4)	<.001	411.5 (410.3 to 412.8)	−0.3 (−0.9 to 0.3)	.28	299.0 (297.5 to 300.5)	0.6 (0.5 to 0.7)	<.001	388.4 (382.4 to 394.5)	0.4 (0.2 to 0.5)	<.001	335.4 (334.3 to 336.5)	0.6 (0.4 to 0.8)	<.001
Children (aged 0-14 y)	—	16.8 (16.7 to 17.0)	0.7 (0.5 to 0.9)	<.001	17.8 (17.6 to 18.0)	0.7 (0.5 to 0.9)	<.001	13.4 (13.0 to 13.7)	1.0 (0.6 to 1.4)	<.001	14.9 (14.4 to 15.5)	1.7 (1.1 to 2.2)	<.001	12.6 (11.4 to 13.9)	0.0 (−1.3 to 1.3)	.97	16.4 (16.1 to 16.7)	0.4 (0.1 to 0.7)	.006
AYA (aged 15-39 y)	—	75.9 (75.6 to 76.1)	0.9 (0.8 to 1.0)	<.001	84.4 (84.0 to 84.7)	0.3 (−1.1 to 1.6)	.71	63.5 (62.9 to 64.1)	0.7 (0.5 to 0.8)	<.001	55.6 (54.8 to 56.4)	1.7 (1.5 to 1.9)	<.001	64.1 (61.5 to 66.7)	5.0 (−0.7 to 11.1)	.09	62.8 (62.4 to 63.3)	2.3 (1.7 to 2.8)	<.001
Males																			
Prostate	1	108.5 (108.3 to 108.7)	0.6 (−3.6 to 5.1)	.77	102.4 (102.2 to 102.7)	0.9 (−3.8 to 5.8)	.71	180.7 (179.7 to 181.7)	−0.3 (−3.4 to 2.9)	.87	57.4 (56.6 to 58.2)	1.0 (−3.0 to 5.1)	.64	75.0 (71.9 to 78.1)	0.5 (−4.5 to 5.7)	.85	95.7 (95.0 to 96.4)	−1.4 (−4.3 to 1.5)	.35
Lung and bronchus	2	68.0 (67.8 to 68.2)	−2.5 (−2.7 to −2.4)	<.001	71.8 (71.6 to 72.0)	−2.3 (−2.4 to −2.2)	<.001	80.8 (80.1 to 81.4)	−3.2 (−3.5 to −2.8)	<.001	44.1 (43.4 to 44.8)	−1.4 (−1.7 to −1.2)	<.001	61.9 (59.0 to 64.8)	−1.6 (−2.4 to −0.9)	<.001	35.8 (35.4 to 36.2)	−2.8 (−3.0 to −2.5)	<.001
Colon and rectum	3	44.6 (44.5 to 44.8)	−1.1 (−1.8 to −0.5)	.002	44.3 (44.1 to 44.4)	−1.2 (−1.6 to −0.7)	<.001	53.7 (53.2 to 54.3)	−2.7 (−2.9 to −2.5)	<.001	36.0 (35.4 to 36.6)	−2.1 (−2.4 to −1.9)	<.001	50.9 (48.4 to 53.4)	−0.8 (−1.4 to −0.2)	.009	42.2 (41.7 to 42.6)	−2.1 (−2.4 to −1.9)	<.001
Urinary bladder	4	35.1 (35.0 to 35.3)	−1.5 (−2.1 to −1.0)	<.001	39.7 (39.5 to 39.8)	−1.5 (−2.2 to −0.8)	.001	20.2 (19.8 to 20.5)	−1.4 (−2.9 to 0.2)	.08	15.4 (15.0 to 15.8)	−0.5 (−0.8 to −0.2)	.001	21.1 (19.4 to 23.0)	1.0 (0.1 to 1.9)	.03	18.8 (18.5 to 19.2)	−1.4 (−1.7 to −1.2)	<.001
Skin melanoma	5	29.1 (29.0 to 29.3)	2.2 (2.0 to 2.4)	<.001	37.4 (37.3 to 37.6)	2.6 (2.5 to 2.8)	<.001	1.2 (1.1 to 1.3)	−0.2 (−1.2 to 0.9)	.72	1.6 (1.5 to 1.7)	−0.3 (−0.9 to 0.3)	.26	10.8 (9.6 to 12.0)	−1.2 (−13.2 to 12.5)	.85	5.1 (5.0 to 5.3)	0.5 (0.0 to 1.0)	.07
Non-Hodgkin lymphoma	6	23.9 (23.8 to 24.0)	−0.6 (−1.6 to 0.3)	.19	25.2 (25.1 to 25.4)	0.1 (−0.1 to 0.3)	.30	18.0 (17.7 to 18.3)	0.2 (0.0 to 0.5)	.04	16.6 (16.2 to 17.0)	0.5 (0.1 to 0.9)	.01	15.9 (14.6 to 17.4)	−1.0 (−2.2 to 0.3)	.11	20.4 (20.1 to 20.7)	0.1 (−0.2 to 0.4)	.47
Kidney and renal pelvis	7	23.2 (23.1 to 23.4)	1.4 (1.0 to 1.7)	<.001	23.7 (23.5 to 23.8)	1.0 (0.8 to 1.2)	<.001	26.8 (26.4 to 27.1)	0.0 (−2.9 to 3.0)	.99	11.7 (11.4 to 12.1)	1.9 (1.3 to 2.6)	<.001	33.1 (31.3 to 35.1)	0.8 (−0.2 to 1.9)	.11	21.4 (21.1 to 21.7)	1.9 (0.9 to 3.0)	.002
Leukemia	8	19.3 (19.2 to 19.4)	−0.5 (−1.5 to 0.5)	.31	20.7 (20.6 to 20.9)	−0.2 (−1.6 to 1.2)	.74	14.8 (14.5 to 15.1)	0.9 (0.4 to 1.3)	.001	10.5 (10.1 to 10.8)	1.0 (0.4 to 1.5)	.002	13.9 (12.6 to 15.3)	0.4 (−0.8 to 1.7)	.45	14.3 (14.1 to 14.6)	0.7 (0.3 to 1.1)	.003
Oral cavity and pharynx	9	18.3 (18.2 to 18.4)	0.9 (0.8 to 1.1)	<.001	20.3 (20.2 to 20.4)	1.6 (1.4 to 1.7)	<.001	14.4 (14.1 to 14.7)	−1.8 (−2.1 to −1.5)	<.001	11.9 (11.5 to 12.2)	0.8 (0.3 to 1.2)	.002	14.6 (13.4 to 15.9)	0.9 (−0.4 to 2.2)	.17	11.1 (10.9 to 11.3)	−0.6 (−1.0 to −0.2)	.009
Pancreas	10	14.9 (14.8 to 15.0)	1.1 (1.0 to 1.2)	<.001	15.1 (15.0 to 15.2)	1.3 (1.2 to 1.4)	<.001	17.8 (17.5 to 18.2)	0.6 (0.3 to 0.9)	.001	10.5 (10.1 to 10.8)	0.6 (0.3 to 1.0)	.003	13.9 (12.6 to 15.3)	2.1 (0.5 to 3.7)	.01	12.1 (11.9 to 12.4)	0.5 (0.2 to 0.8)	.002
Liver and intrahepatic bile duct	11	13.2 (13.1 to 13.3)	1.0 (−0.3 to 2.4)	.13	11.0 (10.9 to 11.1)	1.9 (0.9 to 2.9)	.001	18.4 (18.1 to 18.7)	−0.6 (−3.9 to 2.9)	.73	19.8 (19.4 to 20.3)	−1.0 (−1.3 to −0.6)	<.001	24.1 (22.5 to 25.7)	4.5 (3.3 to 5.8)	<.001	19.8 (19.5 to 20.1)	0.3 (−0.6 to 1.2)	.50
Myeloma	12	9.1 (9.1 to 9.2)	0.7 (−0.1 to 1.5)	.09	8.3 (8.2 to 8.4)	1.1 (0.2 to 1.9)	.02	18.0 (17.6 to 18.3)	2.1 (1.8 to 2.4)	<.001	5.4 (5.1 to 5.6)	2.3 (1.7 to 2.9)	<.001	9.2 (8.1 to 10.4)	4.7 (1.2 to 8.3)	.01	8.4 (8.2 to 8.7)	1.3 (0.8 to 1.8)	<.001
Stomach	13	9.0 (8.9 to 9.0)	−1.3 (−1.9 to −0.8)	<.001	7.6 (7.6 to 7.7)	−1.6 (−2.2 to −1.0)	<.001	13.9 (13.6 to 14.2)	−1.8 (−2.1 to −1.6)	<.001	13.3 (12.9 to 13.7)	−2.8 (−3.3 to −2.4)	<.001	11.6 (10.5 to 12.9)	−3.0 (−3.9 to −2.1)	<.001	12.0 (11.7 to 12.2)	−2.0 (−2.3 to −1.7)	<.001
Esophagus	14	7.9 (7.8 to 8.0)	−0.5 (−1.0 to 0.1)	.08	8.7 (8.7 to 8.8)	−0.6 (−1.0 to −0.2)	.01	6.4 (6.2 to 6.6)	−4.6 (−4.9 to −4.3)	<.001	3.6 (3.4 to 3.8)	−0.9 (−1.8 to 0.0)	.04	8.0 (7.0 to 9.0)	−0.2 (−2.0 to 1.6)	.78	4.9 (4.7 to 5.1)	−1.5 (−2.0 to −1.1)	<.001
Brain and other nervous system	15	7.8 (7.7 to 7.8)	−0.3 (−0.4 to −0.2)	<.001	8.8 (8.8 to 8.9)	0.0 (−0.1 to 0.1)	.80	4.9 (4.7 to 5.0)	0.1 (−0.4 to 0.6)	.74	4.5 (4.3 to 4.7)	3.0 (−1.0 to 7.2)	.14	5.5 (4.7 to 6.3)	0.2 (−1.5 to 2.1)	.77	5.8 (5.6 to 5.9)	−0.7 (−1.0 to −0.5)	<.001
Thyroid	16	7.5 (7.4 to 7.5)	−0.8 (−2.3 to 0.7)	.31	8.3 (8.2 to 8.4)	−0.7 (−2.3 to 0.9)	.39	4.0 (3.9 to 4.2)	−3.1 (−7.6 to 1.7)	0.20	7.5 (7.2 to 7.7)	−0.9 (−4.3 to 2.5)	.56	4.7 (4.1 to 5.5)	3.9 (1.7 to 6.2)	.002	6.3 (6.1 to 6.4)	1.9 (0.3 to 3.5)	.02
Testis	17	5.8 (5.7 to 5.8)	0.5 (0.4 to 0.7)	<.001	7.1 (7.0 to 7.2)	0.5 (0.3 to 0.7)	<.001	1.5 (1.5 to 1.6)	1.0 (0.3 to 1.7)	.01	2.1 (2.0 to 2.2)	2.4 (1.4 to 3.3)	<.001	5.7 (5.0 to 6.4)	1.6 (0.1 to 3.1)	.04	5.1 (5.0 to 5.2)	2.3 (2.0 to 2.7)	<.001
Larynx	18	5.7 (5.6 to 5.7)	−2.3 (−2.5 to −2.2)	<.001	5.7 (5.7 to 5.8)	−2.0 (−2.2 to −1.8)	<.001	8.1 (7.9 to 8.4)	−3.1 (−3.5 to −2.8)	<.001	2.0 (1.9 to 2.2)	−2.5 (−3.8 to −1.3)	.001	5.2 (4.4 to 6.0)	−1.5 (−3.0 to 0.0)	.05	4.7 (4.5 to 4.8)	−3.2 (−3.6 to −2.8)	<.001
Females																			
Breast	1	127.5 (127.3 to 127.8)	0.5 (0.3 to 0.6)	<.001	133.8 (133.5 to 134.1)	0.6 (0.4 to 0.7)	<.001	129.8 (129.1 to 130.5)	0.4 (0.0 to 0.8)	.04	98.7 (97.9 to 99.6)	1.7 (1.4 to 2.0)	<.001	101.6 (98.6 to 104.7)	0.6 (0.1 to 1.0)	.02	96.8 (96.2 to 97.4)	0.4 (0.2 to 0.6)	.001
Lung and bronchus	2	51.5 (51.3 to 51.6)	−1.1 (−1.2 to −0.9)	<.001	57.2 (57.0 to 57.3)	−0.8 (−1.0 to −0.7)	<.001	48.5 (48.1 to 49.0)	−1.7 (−2.0 to −1.3)	<.001	28.5 (28.0 to 29.0)	0.1 (−0.2 to 0.3)	.51	50.2 (48.0 to 52.5)	−0.3 (−0.8 to 0.1)	.15	23.1 (22.8 to 23.4)	−0.7 (−1.0 to −0.5)	<.001
Colon and rectum	3	34.2 (34.0 to 34.3)	−0.9 (−1.2 to −0.5)	<.001	34.2 (34.1 to 34.4)	−0.6 (−0.9 to −0.3)	.002	39.8 (39.5 to 40.2)	−2.0 (−2.8 to −1.2)	<.001	26.1 (25.6 to 26.6)	−0.8 (−2.5 to 0.9)	.30	41.7 (39.7 to 43.7)	−0.7 (−1.2 to −0.3)	.002	29.9 (29.6 to 30.3)	0.3 (−0.7 to 1.2)	.56
Corpus and uterus, NOS	4	27.3 (27.2 to 27.4)	1.3 (1.2 to 1.4)	<.001	27.9 (27.8 to 28.0)	1.1 (1.0 to 1.2)	<.001	27.8 (27.5 to 28.1)	2.4 (2.2 to 2.6)	<.001	20.2 (19.8 to 20.6)	2.3 (2.0 to 2.6)	<.001	24.6 (23.2 to 26.1)	1.4 (0.6 to 2.2)	.002	24.4 (24.1 to 24.7)	2.1 (1.8 to 2.4)	<.001
Thyroid	5	21.7 (21.6 to 21.8)	−2.0 (−3.5 to −0.6)	.007	22.9 (22.8 to 23.0)	−2.2 (−3.9 to −0.5)	.01	13.9 (13.7 to 14.2)	−3.8 (−7.5 to 0.0)	.05	22.3 (21.9 to 22.7)	−1.7 (−3.3 to −0.2)	.03	16.8 (15.6 to 18.0)	4.6 (3.6 to 5.6)	<.001	23.1 (22.8 to 23.3)	−0.2 (−2.8 to 2.4)	.86
Skin melanoma	6	18.0 (17.9 to 18.1)	1.9 (1.6 to 2.1)	<.001	24.5 (24.4 to 24.7)	2.5 (2.2 to 2.8)	<.001	1.0 (0.9 to 1.0)	−0.7 (−1.5 to 0.1)	.10	1.3 (1.2 to 1.4)	−0.2 (−1.4 to 0.9)	.68	6.7 (6.0 to 7.6)	1.7 (0.4 to 3.1)	.02	4.5 (4.4 to 4.6)	2.6 (0.8 to 4.4)	.008
Non-Hodgkin lymphoma	7	16.4 (16.3 to 16.5)	−0.1 (−0.2 to 0.0)	.13	17.1 (17.0 to 17.2)	−0.1 (−0.2 to 0.0)	.11	12.7 (12.5 to 13.0)	0.5 (0.3 to 0.8)	.001	11.2 (10.9 to 11.5)	0.3 (−0.3 to 0.8)	.29	13.9 (12.7 to 15.1)	−0.7 (−1.5 to 0.1)	.07	15.7 (15.4 to 15.9)	0.4 (0.2 to 0.7)	.002
Kidney and renal pelvis	8	11.9 (11.8 to 12.0)	1.6 (0.6 to 2.7)	.007	12.0 (11.9 to 12.1)	1.8 (0.8 to 2.7)	.002	13.6 (13.4 to 13.9)	0.4 (−0.2 to 1.0)	.17	5.5 (5.3 to 5.7)	1.4 (0.6 to 2.2)	.001	18.8 (17.5 to 20.1)	1.5 (0.5 to 2.4)	.005	12.0 (11.8 to 12.2)	1.6 (1.3 to 1.9)	<.001
Leukemia	9	11.7 (11.7 to 11.8)	0.2 (−0.4 to 0.8)	.42	12.4 (12.3 to 12.5)	0.4 (−0.4 to 1.2)	.26	9.6 (9.4 to 9.8)	−0.3 (−1.9 to 1.4)	.73	6.8 (6.5 to 7.0)	1.1 (0.6 to 1.5)	<.001	8.3 (7.4 to 9.2)	0.3 (−1.1 to 1.7)	.68	10.0 (9.8 to 10.2)	1.0 (0.6 to 1.3)	<.001
Pancreas	10	11.4 (11.4 to 11.5)	1.0 (0.9 to 1.1)	<.001	11.3 (11.2 to 11.4)	1.2 (1.0 to 1.3)	<.001	15.0 (14.7 to 15.2)	0.6 (0.4 to 0.9)	<.001	8.8 (8.5 to 9.1)	0.7 (0.3 to 1.1)	.001	10.4 (9.5 to 11.5)	0.2 (−1.0 to 1.5)	.71	10.2 (10.0 to 10.4)	0.8 (0.5 to 1.0)	<.001
Ovary	11	11.2 (11.1 to 11.2)	−1.6 (−1.7 to −1.5)	<.001	11.6 (11.5 to 11.7)	−1.7 (−1.9 to −1.6)	<.001	9.2 (9.0 to 9.4)	−0.9 (−1.2 to −0.6)	<.001	9.4 (9.1 to 9.7)	−2.3 (−4.9 to 0.4)	.09	11.1 (10.1 to 12.2)	−1.0 (−2.5 to 0.4)	.15	10.1 (9.9 to 10.3)	−1.2 (−1.5 to −1.0)	<.001
Urinary bladder	12	8.7 (8.6 to 8.7)	−0.9 (−1.0 to −0.7)	<.001	9.7 (9.7 to 9.8)	−0.7 (−0.8 to −0.5)	<.001	6.8 (6.6 to 6.9)	−0.5 (−0.8 to −0.2)	.004	3.7 (3.6 to 3.9)	−0.7 (−1.5 to 0.1)	.09	5.5 (4.8 to 6.3)	0.9 (−0.8 to 2.7)	.27	4.9 (4.8 to 5.1)	−1.2 (−1.7 to −0.8)	<.001
Cervix uteri	13	7.8 (7.7 to 7.8)	1.1 (−0.3 to 2.5)	.12	7.3 (7.2 to 7.4)	1.2 (−0.5 to 3.0)	.15	9.1 (8.9 to 9.3)	−2.2 (−2.6 to −1.9)	<.001	6.2 (6.0 to 6.4)	0.2 (−0.5 to 0.9)	.50	9.4 (8.5 to 10.3)	−0.2 (−1.1 to 0.8)	.72	9.9 (9.7 to 10.0)	0.2 (−1.5 to 2.0)	.77
Oral cavity and pharynx	14	6.6 (6.5 to 6.6)	0.5 (0.3 to 0.6)	<.001	7.1 (7.1 to 7.2)	0.9 (0.7 to 1.0)	<.001	5.2 (5.1 to 5.4)	−0.7 (−0.9 to −0.5)	<.001	5.3 (5.1 to 5.5)	0.1 (−0.6 to 0.8)	.73	5.9 (5.1 to 6.6)	−0.4 (−2.1 to 1.4)	.66	4.3 (4.2 to 4.5)	0.3 (−0.2 to 0.8)	.26
Myeloma	15	6.0 (5.9 to 6.0)	1.5 (1.1 to 1.9)	<.001	5.1 (5.0 to 5.1)	1.4 (0.9 to 1.8)	<.001	13.2 (13.0 to 13.4)	2.2 (1.9 to 2.6)	<.001	3.3 (3.1 to 3.5)	1.0 (0.3 to 1.7)	.006	6.4 (5.6 to 7.3)	0.2 (−1.5 to 2.0)	.80	5.9 (5.8 to 6.1)	2.0 (1.2 to 2.7)	<.001
Brain and other nervous system	16	5.6 (5.5 to 5.6)	−0.5 (−0.8 to −0.3)	<.001	6.4 (6.3 to 6.4)	−0.3 (−0.6 to −0.1)	.02	3.6 (3.5 to 3.7)	−0.3 (−0.7 to 0.2)	.22	3.2 (3.1 to 3.4)	−4.5 (−6.5 to −2.4)	<.001	3.5 (3.0 to 4.1)	7.9 (−4.3 to 21.7)	.17	4.5 (4.4 to 4.6)	−0.5 (−0.9 to −0.1)	.01
Stomach	17	4.7 (4.7 to 4.7)	0.1 (−0.3 to 0.5)	.49	3.5 (3.5 to 3.6)	0.0 (−0.5 to 0.5)	.95	7.5 (7.4 to 7.7)	−1.2 (−1.5 to −0.9)	<.001	7.8 (7.6 to 8.1)	−2.5 (−2.9 to −2.0)	<.001	7.0 (6.2 to 7.8)	−0.9 (−2.5 to 0.7)	.24	7.7 (7.5 to 7.9)	−1.1 (−1.5 to −0.8)	<.001
Liver and intrahepatic bile duct	18	4.7 (4.6 to 4.7)	2.3 (0.9 to 3.7)	.001	3.9 (3.9 to 4.0)	3.9 (3.7 to 4.2)	<.001	5.6 (5.5 to 5.8)	3.3 (2.7 to 3.9)	<.001	7.3 (7.1 to 7.6)	−1.1 (−1.7 to −0.6)	.001	9.8 (8.9 to 10.8)	2.5 (1.0 to 4.0)	.003	7.7 (7.5 to 7.9)	2.3 (1.9 to 2.7)	<.001
Children (aged 0-14 y)																			
Leukemia	—	5.2 (5.1 to 5.3)	0.7 (0.4 to 1.0)	<.001	5.2 (5.1 to 5.4)	0.5 (0.2 to 0.8)	.001	3.2 (3.1 to 3.4)	1.3 (0.7 to 1.9)	<.001	5.2 (4.9 to 5.5)	0.6 (−0.1 to 1.4)	.09	4.8 (4.0 to 5.7)	7.1 (0.8 to 13.7)	.03	6.2 (6.1 to 6.4)	0.7 (0.2 to 1.2)	.007
Brain and other nervous system	—	3.8 (3.7 to 3.9)	0.7 (0.5 to 1.0)	<.001	4.3 (4.2 to 4.4)	0.9 (0.6 to 1.3)	<.001	3.0 (2.9 to 3.2)	1.5 (0.8 to 2.2)	<.001	2.8 (2.6 to 3.1)	1.1 (0.0 to 2.2)	.05	2.4 (1.9 to 3.0)	0.0 (−2.2 to 2.1)	.97	3.0 (2.9 to 3.2)	0.1 (−0.3 to 0.4)	.71
Lymphoma	—	1.6 (1.6 to 1.7)	0.8 (0.4 to 1.2)	.001	1.7 (1.6 to 1.8)	0.9 (0.3 to 1.5)	.01	1.6 (1.5 to 1.7)	1.3 (0.4 to 2.2)	.006	1.5 (1.4 to 1.7)	1.3 (−0.2 to 2.8)	.09	1.2 (0.8 to 1.6)	1.3 (−3.3 to 6.2)	.56	1.5 (1.4 to 1.6)	0.2 (−0.4 to 0.7)	.54
AYA (aged 15-39 y)																			
Female breast	—	22.6 (22.4 to 22.8)	1.1 (0.5 to 1.6)	.001	23.3 (23.0 to 23.5)	0.6 (0.4 to 0.8)	<.001	27.1 (26.5 to 27.7)	0.4 (0.1 to 0.6)	.004	19.9 (19.2 to 20.5)	0.8 (0.3 to 1.3)	.003	17.7 (15.7 to 19.8)	0.7 (−1.0 to 2.4)	.40	17.8 (17.5 to 18.2)	1.7 (0.5 to 2.9)	.01
Thyroid	—	12.1 (12.0 to 12.2)	−1.2 (−3.2 to 0.9)	.27	13.8 (13.7 to 14.0)	−1.4 (−4.0 to 1.4)	.33	5.6 (5.4 to 5.8)	−4.9 (−9.9 to 0.5)	.07	12.1 (11.8 to 12.5)	−0.3 (−2.4 to 1.9)	.76	9.0 (8.0 to 10.0)	3.7 (2.6 to 4.8)	<.001	10.5 (10.3 to 10.7)	1.0 (−3.9 to 6.2)	.70
Testis	—	11.0 (10.9 to 11.2)	0.8 (0.6 to 0.9)	<.001	13.4 (13.3 to 13.6)	0.5 (0.3 to 0.7)	<.001	2.6 (2.4 to 2.8)	0.7 (−0.3 to 1.8)	.16	4.1 (3.8 to 4.4)	3.0 (1.9 to 4.2)	<.001	10.9 (9.5 to 12.4)	0.4 (−0.8 to 1.6)	.47	11.2 (10.9 to 11.5)	2.9 (2.5 to 3.3)	<.001
Lymphoma	—	7.7 (7.6 to 7.7)	−0.4 (−0.5 to −0.3)	<.001	8.2 (8.1 to 8.3)	−0.4 (−0.6 to −0.3)	<.001	8.3 (8.1 to 8.6)	−0.9 (−1.3 to −0.4)	.002	5.3 (5.1 to 5.5)	2.0 (1.4 to 2.6)	<.001	4.4 (3.7 to 5.1)	0.7 (−1.5 to 3.0)	.49	6.2 (6.0 to 6.3)	0.0 (−0.3 to 0.2)	.85
Skin melanoma	—	6.7 (6.7 to 6.8)	−0.7 (−1.1 to −0.4)	.001	10.6 (10.5 to 10.8)	0.1 (−0.3 to 0.6)	.52	0.2 (0.2 to 0.3)	−1.4 (−3.3 to 0.5)	.13	0.5 (0.4 to 0.5)	−2.1 (−3.6 to −0.5)	.01	2.5 (2.0 to 3.0)	−0.7 (−2.6 to 1.3)	.45	1.2 (1.2 to 1.3)	−1.5 (−2.2 to −0.8)	<.001
Colon and rectum	—	4.8 (4.8 to 4.9)	5.5 (4.1 to 7.0)	<.001	5.3 (5.2 to 5.4)	6.1 (4.2 to 8.0)	<.001	4.8 (4.6 to 5.0)	3.5 (2.4 to 4.7)	<.001	3.3 (3.2 to 3.5)	1.2 (0.2 to 2.1)	.02	5.8 (5.0 to 6.6)	2.6 (0.4 to 4.9)	.02	3.8 (3.7 to 3.9)	9.0 (5.7 to 12.5)	<.001

a The following National Program of Cancer Registries and Surveillance, Epidemiology, and End Results areas are reported by the North American Association of Central Cancer Registries as meeting high-quality incidence data standards for 2013-2017 (49 states, District of Columbia, and 1 territory): Alabama, Alaska, Arizona, Arkansas, California, Colorado, Connecticut, District of Columbia, Delaware, Florida, Georgia, Hawaii, Idaho, Illinois, Indiana, Iowa, Kansas, Kentucky, Louisiana, Maine, Maryland, Massachusetts, Michigan, Minnesota, Mississippi, Missouri, Montana, Nebraska, New Hampshire, New Jersey, New Mexico, New York, North Carolina, North Dakota, Ohio, Oklahoma, Oregon, Pennsylvania, Puerto Rico, Rhode Island, South Carolina, South Dakota, Tennessee, Texas, Utah, Vermont, Virginia, Washington, West Virginia, Wisconsin, and Wyoming. AAPC = average annual percent change; AI/AN = American Indian/Alaska Native; APC = annual percent change; API = Asian/Pacific Islander; CI = confidence interval; NOS = not otherwise specified; PRCDA = Indian Health Services Purchased/Referred Care Delivery Area.

b Cancers are sorted in descending order according to sex-specific rates for all racial/ethnic groups combined. More than 15 cancers may appear under males and females to include the top 15 cancers in every racial/ethnic group.

c Racial/ethnic groups are mutually exclusive. Data for non-Hispanic AI/AN persons are restricted to PRCDA counties (excluding Minnesota).

d Rates are per 100 000 population, adjusted for potential delays in reporting and are age standardized to the 2000 US standard population (19 age groups, Census P25–1130).

e AAPC is the average annual percent change and is a weighted average of the APCs over the fixed interval 2013-2017 using the underlying Joinpoint model for the period of 2001-2017. Joinpoint models with up to 3 joinpoints are based on rates per 100 000 population age standardized to the 2000 US standard population (19 age groups, Census P25–1130). Joinpoint Regression Program was used (version 4.8.0.1; Statistical Research and Applications Branch, National Cancer Institute; April 2020).

**Table 2.** Joinpoint incidence trends (2001-2017) for the most common cancers, all ages, all racial/ethnic groups combined by sex and age group, for areas in the United States with high-quality incidence data^a^

Sex and cancer site or type^b^	Trends in 2001-2017													
	1st segment			2nd segment			3rd segment			4th segment			AAPC^c^	
	Years	APC (95% CI)	*P*	Years	APC (95% CI)	*P*	Years	APC (95% CI)	*P*	Years	APC (95% CI)	*P*	2013-2017 (95% CI)	*P*
All sites														
Both sexes combined	2001-2004	−1.2 (−2.3 to −0.1)	.04	2004-2007	0.6 (−1.7 to 2.9)	.55	2007-2013	−1.1 (−1.6 to −0.6)	.002	2013-2017	0.0 (−0.7 to 0.7)	.98	0.0 (−0.7 to 0.7)	.98
Males	2001-2004	−1.7 (−3.4 to 0.1)	.06	2004-2007	0.5 (−2.9 to 4.0)	.75	2007-2013	−2.2 (−3.0 to −1.5)	<.001	2013-2017	−0.3 (−1.4 to 0.7)	.48	−0.3 (−1.4 to 0.7)	.48
Females	2001-2003	−1.1 (−3.0 to 0.8)	.23	2003-2017	0.2 (0.1 to 0.2)	.002	–	–	–	–	–	–	0.2 (0.1 to 0.2)	.002
Children (aged 0-14 y)	2001-2017	0.7 (0.5 to 0.9)	<.001	–	–	–	–	–	–	–	–	–	0.7 (0.5 to 0.9)	<.001
AYA (aged 15-39 y)	2001-2017	0.9 (0.8 to 1.0)	<.001	–	–	–	–	–	–	–	–	–	0.9 (0.8 to 1.0)	<.001
Top 18 cancers for males														
Prostate	2001-2004	−5.1 (−11.5 to 1.6)	.11	2004-2007	3.0 (−10.2 to 18.1)	.61	2007-2014	−6.6 (−8.8 to −4.3)	<.001	2014-2017	3.2 (−4.0 to 10.9)	.33	0.6 (−3.6 to 5.1)	.77
Lung and bronchus	2001-2007	−1.6 (−1.8 to −1.3)	<.001	2007-2017	−2.5 (−2.7 to −2.4)	<.001	–	–	–	–	–	–	−2.5 (−2.7 to −2.4)	<.001
Colon and rectum	2001-2012	−3.2 (−3.4 to −3.1)	<.001	2012-2017	−1.1 (−1.8 to −0.5)	.002	–	–	–	–	–	–	−1.1 (−1.8 to −0.5)	.002
Urinary bladder	2001-2004	0.2 (−0.8 to 1.2)	.61	2004-2013	−0.7 (−0.9 to −0.5)	<.001	2013-2017	−1.5 (−2.1 to −1.0)	<.001	–	–	–	−1.5 (−2.1 to −1.0)	<.001
Skin melanoma	2001-2017	2.2 (2.0 to 2.4)	<.001	–	–	–	–	–	–	–	–	–	2.2 (2.0 to 2.4)	<.001
Non−Hodgkin lymphoma	2001-2014	0.3 (0.1 to 0.4)	.004	2014-2017	−0.9 (−2.3 to 0.5)	.17	–	–	–	–	–	–	−0.6 (−1.6 to 0.3)	.19
Kidney and renal pelvis	2001-2007	3.0 (2.5 to 3.5)	<.001	2007-2011	0.1 (−1.1 to 1.3)	.89	2011-2017	1.4 (1.0 to 1.7)	<.001	–	–	–	1.4 (1.0 to 1.7)	<.001
Leukemia	2001-2007	−0.2 (−0.8 to 0.3)	.37	2007-2014	2.0 (1.4 to 2.5)	<.001	2014-2017	−1.3 (−2.8 to 0.2)	.07	–	–	–	−0.5 (−1.5 to 0.5)	.31
Oral cavity and pharynx	2001-2017	0.9 (0.8 to 1.1)	<.001	–	–	–	–	–	–	–	–	–	0.9 (0.8 to 1.1)	<.001
Pancreas	2001-2017	1.1 (1.0 to 1.2)	<.001	–	–	–	–	–	–	–	–	–	1.1 (1.0 to 1.2)	<.001
Liver and intra hepatic bile duct	2001-2009	4.6 (4.1 to 5.0)	<.001	2009-2015	2.9 (2.1 to 3.6)	<.001	2015-2017	−0.8 (−3.7 to 2.3)	.58	–	–	–	1.0 (−0.3 to 2.4)	.13
Myeloma	2001-2007	0.6 (0.1 to 1.2)	.03	2007-2013	3.0 (2.3 to 3.6)	<.001	2013-2017	0.7 (−0.1 to 1.5)	.09	–	–	–	0.7 (−0.1 to 1.5)	.09
Stomach	2001-2008	−1.8 (−2.3 to −1.4)	<.001	2008-2011	0.6 (−2.6 to 3.9)	.69	2011-2017	−1.3 (−1.9 to −0.8)	<.001	–	–	–	−1.3 (−1.9 to −0.8)	<.001
Esophagus	2001-2008	0.3 (−0.2 to 0.7)	.23	2008-2011	−2.9 (−6.1 to 0.4)	.08	2011-2017	−0.5 (−1.0 to 0.1)	.08	–	–	–	−0.5 (−1.0 to 0.1)	.08
Brain and other nervous system	2001-2017	−0.3 (−0.4 to −0.2)	<.001	–	–	–	–	–	–	–	–	–	−0.3 (−0.4 to −0.2)	<.001
Thyroid	2001-2009	7.0 (6.3 to 7.7)	<.001	2009-2014	2.3 (0.8 to 3.8)	.008	2014-2017	−1.8 (−4.0 to 0.5)	.11	–	–	–	−0.8 (−2.3 to 0.7)	.31
Testis	2001-2017	0.5 (0.4 to 0.7)	<.001	–	–	–	–	–	–	–	–	–	0.5 (0.4 to 0.7)	<.001
Larynx	2001-2017	−2.3 (−2.5 to −2.2)	<.001	–	–	–	–	–	–	–	–	–	−2.3 (−2.5 to −2.2)	<.001
Top 18 cancers for females														
Breast	2001-2004	−2.9 (−4.3 to −1.5)	.001	2004-2017	0.5 (0.3 to 0.6)	<.001	–	–	–	–	–	–	0.5 (0.3 to 0.6)	<.001
Lung and bronchus	2001-2006	0.6 (0.2 to 1.1)	.01	2006-2017	−1.1 (−1.2 to −0.9)	<.001	–	–	–	–	–	–	−1.1 (−1.2 to −0.9)	<.001
Colon and rectum	2001-2008	−2.5 (−2.7 to −2.2)	<.001	2008-2011	−4.0 (−5.9 to −2.1)	.001	2011-2017	−0.9 (−1.2 to −0.5)	<.001	–	–	–	−0.9 (−1.2 to −0.5)	<.001
Corpus and uterus, NOS	2001-2003	−2.2 (−4.4 to 0.2)	.07	2003-2017	1.3 (1.2 to 1.4)	<.001	–	–	–	–	–	–	1.3 (1.2 to 1.4)	<.001
Thyroid	2001-2009	7.4 (6.9 to 7.9)	<.001	2009-2015	1.7 (0.9 to 2.4)	.001	2015-2017	−5.6 (−8.8 to −2.4)	.004	–	–	–	−2.0 (−3.5 to −0.6)	0.007
Melanoma of the skin	2001-2017	1.9 (1.6 to 2.1)	<.001	–	–	–	–	–	–	–	–	–	1.9 (1.6 to 2.1)	<.001
Non-Hodgkin lymphoma	2001-2017	−0.1 (−0.2 to 0.0)	.13	–	–	–	–	–	–	–	–	–	−0.1 (−0.2 to 0.0)	.13
Kidney and renal pelvis	2001-2006	4.0 (3.1 to 4.9)	<.001	2006-2013	0.3 (−0.3 to 0.9)	.32	2013-2017	1.6 (0.6 to 2.7)	.007	–	–	–	1.6 (0.6 to 2.7)	.007
Leukemia	2001-2008	0.2 (−0.3 to 0.7)	.36	2008-2011	3.3 (−0.4 to 7.2)	.07	2011-2017	0.2 (−0.4 to 0.8)	.42	–	–	–	0.2 (−0.4 to 0.8)	.42
Pancreas	2001-2017	1.0 (0.9 to 1.1)	<.001	–	–	–	–	–	–	–	–	–	1.0 (0.9 to 1.1)	<.001
Ovary	2001-2017	−1.6 (−1.7 to −1.5)	<.001	–	–	–	–	–	–	–	–	–	−1.6 (−1.7 to −1.5)	<.001
Urinary bladder	2001-2017	−0.9 (−1.0 to −0.7)	<.001	–	–	–	–	–	–	–	–	–	−0.9 (−1.0 to −0.7)	<.001
Cervix uteri	2001-2003	−3.8 (−7.8 to 0.4)	.07	2003-2013	−1.1 (−1.5 to −0.7)	<.001	2013-2017	1.1 (−0.3 to 2.5)	.12	–	–	–	1.1 (−0.3 to 2.5)	.12
Oral cavity and pharynx	2001-2017	0.5 (0.3 to 0.6)	<.001	–	–	–	–	–	–	–	–	–	0.5 (0.3 to 0.6)	<.001
Myeloma	2001-2007	0.2 (−0.3 to 0.6)	.41	2007-2011	3.4 (2.2 to 4.7)	<.001	2011-2017	1.5 (1.1 to 1.9)	<.001	–	–	–	1.5 (1.1 to 1.9)	<.001
Brain and other nervous system	2001-2005	0.8 (−0.4 to 2.0)	.17	2005-2017	−0.5 (−0.8 to −0.3)	<.001	–	–	–	–	–	–	−0.5 (−0.8 to −0.3)	<.001
Stomach	2001-2008	−1.1 (−1.7 to −0.5)	.001	2008-2017	0.1 (−0.3 to 0.5)	.49	–	–	–	–	–	–	0.1 (−0.3 to 0.5)	.49
Liver and intra hepatic bile duct	2001-2014	3.9 (3.6 to 4.2)	<.001	2014-2017	1.8 (−0.2 to 3.8)	.08	–	–	–	–	–	–	2.3 (0.9 to 3.7)	.001
Children (aged 0-14 y)														
Leukemia	2001-2017	0.7 (0.4 to 1.0)	<.001	–	–	–	–	–	–	–	–	–	0.7 (0.4 to 1.0)	<.001
Brain and other nervous system	2001-2017	0.7 (0.5 to 1.0)	<.001	–	–	–	–	–	–	–	–	–	0.7 (0.5 to 1.0)	<.001
Lymphoma	2001-2017	0.8 (0.4 to 1.2)	.001	–	–	–	–	–	–	–	–	–	0.8 (0.4 to 1.2)	.001
AYA (aged 15-39 y)														
Female breast	2001-2010	0.0 (−0.3 to 0.4)	.92	2010-2017	1.1 (0.5 to 1.6)	.001	–	–	–	–	–	–	1.1 (0.5 to 1.6)	.001
Thyroid	2001-2006	5.0 (3.5 to 6.5)	<.001	2006-2009	7.4 (1.5 to 13.6)	.02	2009-2015	2.5 (1.3 to 3.7)	.002	2015-2017	−4.7 (−9.4 to 0.3)	.06	−1.2 (−3.2 to 0.9)	.27
Testis	2001-2017	0.8 (0.6 to 0.9)	<.001	–	–	–	–	–	–	–	–	–	0.8 (0.6 to 0.9)	<.001
Lymphoma	2001-2017	−0.4 (−0.5 to −0.3)	<.001	–	–	–	–	–	–	–	–	–	−0.4 (−0.5 to −0.3)	<.001
Melanoma of the skin	2001-2017	−0.7 (−1.1 to −0.4)	.001	–	–	–	–	–	–	–	–	–	−0.7 (−1.1 to −0.4)	.001
Colon and rectum	2001-2011	1.8 (1.1 to 2.6)	<.001	2011-2017	5.5 (4.1 to 7.0)	<.001	–	–	–	–	–	–	5.5 (4.1 to 7.0)	<.001

a The following National Program of Cancer Registries and Surveillance, Epidemiology, and End Results areas are reported by the North American Association of Central Cancer Registries as meeting high-quality incidence data standards for 2001-2017 (46 states): Alabama, Alaska, Arizona, Arkansas, California, Colorado, Connecticut, Delaware, Florida, Georgia, Hawaii, Idaho, Illinois, Indiana, Iowa, Kansas, Kentucky, Louisiana, Maine, Maryland, Massachusetts, Michigan, Minnesota, Missouri, Montana, Nebraska, New Hampshire, New Jersey, New Mexico, New York, North Carolina, North Dakota, Ohio, Oklahoma, Oregon, Pennsylvania, Rhode Island, South Carolina, South Dakota, Texas, Utah, Vermont, Washington, West Virginia, Wisconsin, and Wyoming. Joinpoint models with up to 3 joinpoints are based on rates per 100 000 population, adjusted for potential delays in reporting, and are age standardized to the 2000 US standard population (19 age groups, Census P25–1130). Joinpoint Regression Program was used (version 4.8.0.1; Statistical Research and Applications Branch, National Cancer Institute; April 2020). AAPC = average annual percent change; APC = annual percent change; CI = confidence interval; NOS = not otherwise specified.

b Cancers are listed in descending rank order of sex-specific, age-adjusted incidence rates for 2013-2017 for all racial and ethnic groups combined. More than 15 cancers may appear under males and females to include the top 15 cancers in each racial and ethnic group.

c AAPC is the average annual percent change and is a weighted average of the APCs over the fixed interval 2013-2017 using the underlying Joinpoint model for the period of 2001-2017.

**Figure 2.**
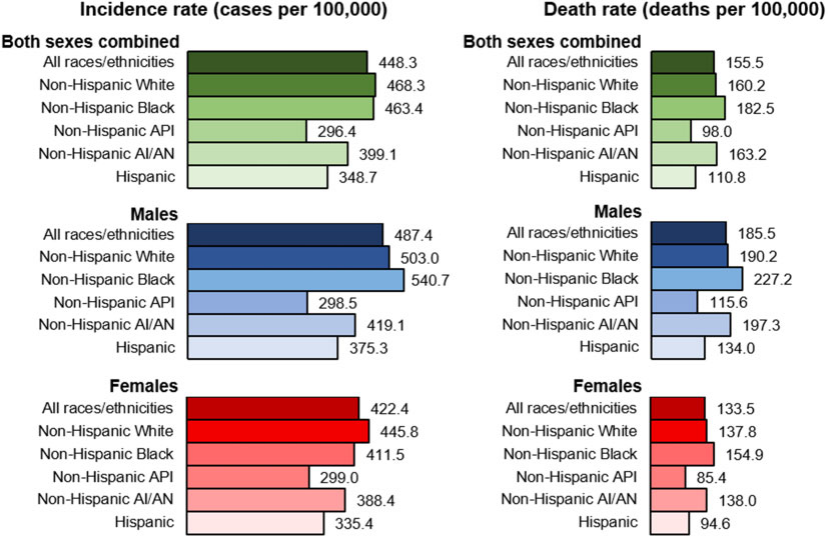
Age-standardized, delay-adjusted overall cancer incidence rates (2013-2017) and age-standardized overall cancer death rates (2014-2018), all cancer sites combined, all ages, by sex and racial and ethnic group. Racial/ethnic groups are mutually exclusive. Data for non-Hispanic American Indian or Alaska Native (AI/AN) are restricted to counties with Indian Health Service Purchased/Referred Care Delivery Areas. API = Asian or Pacific Islander.

By racial/ethnic group, overall cancer incidence rates in the most recent 5 years (2013-2017) were stable among White males and Black females; decreased among Black, API, AI/AN, and Hispanic males; and increased among White, API, AI/AN, and Hispanic females (Table 1). The overall cancer incidence rate was similar among White persons and Black persons, and incidence rates in these 2 groups were higher than rates in other racial/ethnic groups (Figure 2). The overall cancer incidence rate was higher among males than females in every racial/ethnic group, except API, where the rates were similar. Overall incidence rates were higher among Black males than White males and higher among White females than Black females.

**Figure 3.**
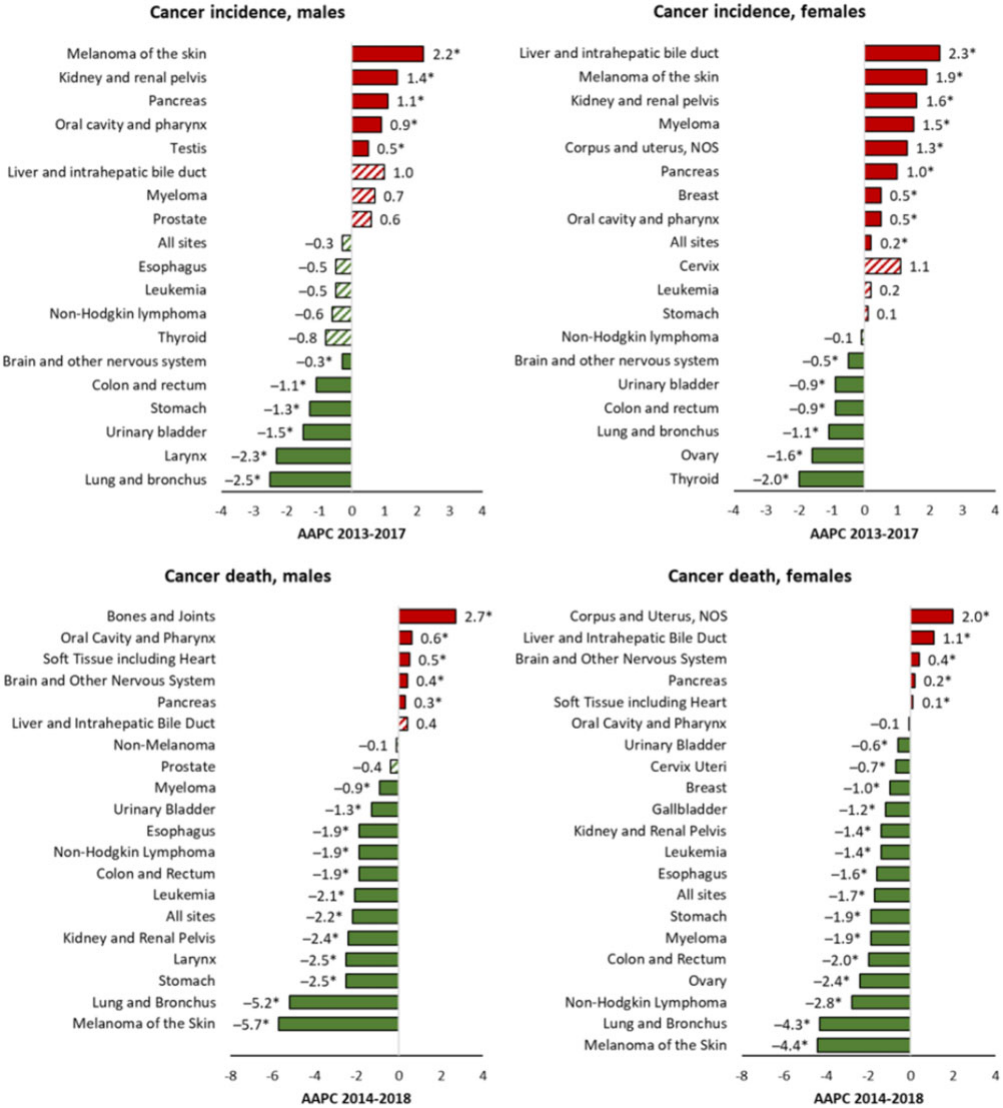
Average annual percent change (AAPC) in age-standardized, delay-adjusted incidence rates for 2013-2017 for all sites and the 18 most common cancers in men and women and age-standardized death rates for 2014-2018 for the 19 most common cancer deaths in men and the 20 most common cancer deaths in women, all ages, all races or ethnicities combined, by sex. The AAPC was a weighted average of the annual percent change (APCs) over the fixed 5-year interval (incidence, 2013-2017; mortality, 2014-2018) using the underlying joinpoint regression model, which allowed up to 3 different APCs, for the 17-year period 2001-2017 for incidence and 18-year period 2001-2018 for mortality. ^a^AAPCs were statistically significantly different from zero (2-sided *P *<* *.05), using a *t* test when the AAPC laid entirely within the last joinpoint segment and a z-test when the last joinpoint fell within the last 5 years of data, and are depicted as **solid-colored bars**; AAPCs with **hash marks** were not statistically significantly different from zero (stable). NOS = not otherwise specified.

During 2013-2017, incidence rates among males increased for 5 of the 18 most common cancers: melanoma, kidney and renal pelvis (kidney), pancreas, oral cavity and pharynx, and testis; were stable for 7 cancers: liver and intrahepatic bile duct (liver), myeloma, prostate, esophagus, leukemia, non-Hodgkin lymphoma, and thyroid; and decreased for 6 cancers: lung and bronchus (lung), larynx, urinary bladder (bladder), stomach, colon and rectum (colorectum); and brain and other nervous systems (ONS) (Figure 3; Table 1). Trends for the 3 most common cancers among males were similar by racial/ethnic group: prostate cancer incidence rates were stable, whereas lung and colorectal cancer incidence rates decreased in all racial/ethnic groups, with greater declines among Black males (Table 1). Incidence rates for the fourth-most common cancer (bladder) declined in White, API, and Hispanic males, were stable among Black males, and increased among AI/AN males.

Among females, incidence rates increased during 2013-2017 for 8 of the 18 most common cancers: liver, melanoma, kidney, myeloma, corpus and uterus, not otherwise specified (uterus), pancreas, breast, and oral cavity and pharynx; were stable for 4 cancers: cervix, leukemia, stomach, and non-Hodgkin lymphoma; and decreased for 6 cancers: thyroid, ovary, lung, colorectum, bladder, and brain and ONS (Figure 3; Table 1). However, liver cancer incidence rates among females stabilized during 2014-2017 (Table 2). For the 4 most common cancers among females, during 2013-2017, breast and uterine cancer incidence rates increased in every racial/ethnic group, whereas lung cancer incidence rates decreased among White, Black, and Hispanic females but were stable among API and AI/AN females. Colorectal cancer incidence rates decreased among White, Black, and AI/AN females but were stable among API and Hispanic females (Table 1). Similar to trends among males, declines in lung and colorectal cancer incidence rates were greater in Black females than females in other racial/ethnic groups.

### Cancer Death Rates and Trends

The overall cancer death rate (per 100 000 population) in 2014-2018 was 155.5, with a higher rate in males (185.5) than in females (133.5) (Figure 2; Table 3). During the most recent 5 years (2014-2018), cancer death rates decreased 1.9% (95% CI = 1.6% to 2.3%) per year on average in both sexes combined, 2.2% (95% CI = 1.9% to 2.5%) among males, and 1.7% (95% CI = 1.4% to 2.1%) among females. Long-term trends in cancer death rates during 2001-2018 show that average declines accelerated from −1.8% (95% CI = −1.8% to −1.7%) per year during 2001-2015 to −2.3% (95% CI = −3.1% to −1.6%) per year during 2015-2018 among males and from −1.4% (95% CI = −1.4% to −1.3%) per year during 2001-2016 to −2.1% (95% CI = −2.9% to −1.2%) per year during 2016-2018 among females (Figure 1; Table 4).

**Table 3.** Age-standardized death rates and fixed-interval trends (2014-2018) for the most common causes of cancer death, all ages, by sex, age group, and racial/ethnic group, United States^a^

Sex and cancer site or type^b^	All racial/ethnic groups combined^c^				Non-Hispanic White^c^			Non-Hispanic Black^c^			Non-Hispanic API^c^			Non-Hispanic AI/AN (PRCDA)^c^			Hispanic^c^		
	Rank	Rate^d^ (95% CI)	AAPC^e^ (95% CI)	*P*	Rate^d^ (95% CI)	AAPC^e^ (95% CI)	*P*	Rate^d^ (95% CI)	AAPC^e^ (95% CI)	*P*	Rate^d^ (95% CI)	AAPC^e^ (95% CI)	*P*	Rate^d^ (95% CI)	AAPC^e^ (95% CI)	*P*	Rate^d^ (95% CI)	AAPC^e^ (95% CI)	*P*
All sites																			
Both sexes combined	—	155.5 (155.3 to 155.6)	−1.9 (−2.3 to −1.6)	<.001	160.2 (160.0 to 160.4)	−1.8 (−2.1 to −1.5)	<.001	182.5 (181.9 to 183.1)	−2.0 (−2.1 to −2.0)	<.001	98.0 (97.4 to 98.7)	−2.1 (−2.9 to −1.2)	<.001	163.2 (160.1 to 166.3)	−0.7 (−1.0 to −0.4)	<.001	110.8 (110.3 to 111.4)	−1.2 (−1.3 to −1.2)	<.001
Males	—	185.5 (185.2 to 185.8)	−2.2 (−2.5 to −1.9)	<.001	190.2 (189.8 to 190.5)	−2.0 (−2.3 to −1.7)	<.001	227.2 (226.1 to 228.3)	−2.6 (−2.7 to −2.6)	<.001	115.6 (114.5 to 116.7)	−1.6 (−1.8 to −1.5)	<.001	197.3 (192.0 to 202.7)	−0.6 (−1.0 to −0.2)	.009	134.0 (133.1 to 134.9)	−1.6 (−1.7 to −1.5)	<.001
Females	—	133.5 (133.3 to 133.7)	−1.7 (−2.1 to −1.4)	<.001	137.8 (137.5 to 138.1)	−1.6 (−2.0 to −1.3)	<.001	154.9 (154.1 to 155.6)	−1.6 (−1.6 to −1.5)	<.001	85.4 (84.6 to 86.2)	−1.0 (−1.2 to −0.9)	<.001	138.0 (134.3 to 141.9)	−0.9 (−1.2 to −0.7)	<.001	94.6 (93.9 to 95.2)	−1.0 (−1.0 to −0.9)	<.001
Children (aged 0-14 y)	—	2.1 (2.0 to 2.1)	−1.4 (−1.7 to −1.1)	<.001	2.1 (2.0 to 2.1)	−1.4 (−1.7 to −1.1)	<.001	2.1 (2.0 to 2.3)	−1.1 (−1.7 to −0.6)	.001	1.8 (1.6 to 2.0)	−1.6 (−3.1 to −0.1)	.04	2.6 (2.0 to 3.4)	–^f^	–	2.0 (1.9 to 2.1)	−1.7 (−2.1 to −1.2)	<.001
AYA (aged 15-39 y)	—	8.9 (8.8 to 9.0)	−0.9 (−1.1 to −0.7)	<.001	8.8 (8.7 to 9.0)	−1.1 (−1.4 to −0.9)	<.001	11.4 (11.1 to 11.6)	−1.1 (−1.5 to −0.8)	<.001	6.8 (6.5 to 7.1)	−0.9 (−1.3 to −0.4)	.001	10.7 (9.5 to 12.0)	−0.8 (−2.2 to 0.8)	.31	8.1 (7.9 to 8.3)	1.0 (0.5 to 1.4)	.002
Males																			
Lung and bronchus	1	46.9 (46.7 to 47.0)	−5.2 (−5.6 to −4.9)	<.001	49.4 (49.3 to 49.6)	−4.9 (−5.2 to −4.6)	<.001	57.0 (56.5 to 57.6)	−5.8 (−6.2 to −5.4)	<.001	28.2 (27.7 to 28.8)	−5.0 (−7.1 to −3.0)	<.001	44.8 (42.3 to 47.4)	−4.1 (−6.5 to −1.6)	.003	23.0 (22.6 to 23.4)	−5.9 (−7.8 to −3.9)	<.001
Prostate	2	19.0 (18.9 to 19.1)	−0.4 (−1.2 to 0.5)	.39	17.9 (17.8 to 18.1)	0.0 (−0.9 to 0.8)	.92	38.3 (37.8 to 38.8)	−1.3 (−3.7 to 1.2)	.29	8.8 (8.5 to 9.1)	−1.9 (−2.5 to −1.4)	<.001	21.0 (19.1 to 23.0)	−1.3 (−2.3 to −0.3)	.01	15.6 (15.3 to 16.0)	−0.9 (−1.9 to 0.1)	.08
Colon and rectum	3	16.3 (16.2 to 16.4)	−1.9 (−2.3 to −1.5)	<.001	16.1 (16.0 to 16.2)	−1.7 (−2.1 to −1.3)	<.001	23.2 (22.8 to 23.5)	−2.6 (−2.8 to −2.4)	<.001	11.3 (11.0 to 11.7)	−2.0 (−2.4 to −1.7)	<.001	22.1 (20.4 to 23.9)	−0.1 (−1.2 to 1.0)	.88	14.0 (13.7 to 14.3)	−1.6 (−1.8 to −1.4)	<.001
Pancreas	4	12.7 (12.6 to 12.8)	0.3 (0.2 to 0.4)	<.001	13.0 (12.9 to 13.1)	0.4 (0.3 to 0.5)	<.001	15.4 (15.1 to 15.7)	−0.1 (−0.3 to 0.1)	.16	8.2 (7.9 to 8.5)	0.0 (−0.4 to 0.4)	.96	11.8 (10.6 to 13.2)	0.2 (−1.2 to 1.5)	.81	9.5 (9.2 to 9.7)	0.0 (−0.4 to 0.4)	.89
Liver and intrahepatic bile duct	5	9.7 (9.6 to 9.7)	0.4 (−0.3 to 1.1)	.24	8.4 (8.4 to 8.5)	0.6 (0.0 to 1.1)	.05	13.4 (13.1 to 13.6)	−1.1 (−2.3 to 0.0)	.06	13.2 (12.8 to 13.5)	−3.5 (−4.3 to −2.8)	<.001	17.1 (15.6 to 18.6)	2.7 (1.7 to 3.7)	<.001	13.3 (13.0 to 13.5)	0.1 (−1.1 to 1.2)	.92
Leukemia	6	8.4 (8.3 to 8.4)	−2.1 (−3.4 to −0.8)	.001	8.9 (8.8 to 9.0)	−2.1 (−2.6 to −1.6)	<.001	7.0 (6.8 to 7.2)	−1.7 (−2.1 to −1.4)	<.001	4.7 (4.5 to 4.9)	−0.6 (−1.4 to 0.1)	.09	6.4 (5.5 to 7.4)	0.0 (−1.7 to 1.7)	.96	5.6 (5.5 to 5.8)	−1.0 (−1.5 to −0.6)	<.001
Urinary bladder	7	7.4 (7.3 to 7.4)	−1.3 (−2.0 to −0.6)	.001	8.2 (8.1 to 8.2)	−1.5 (−3.0 to −0.1)	.04	5.4 (5.2 to 5.6)	−0.3 (−0.7 to 0.2)	.20	2.8 (2.7 to 3.0)	−0.3 (−1.2 to 0.5)	.38	4.4 (3.6 to 5.3)	–^f^	–	3.8 (3.7 to 4.0)	−0.6 (−1.2 to 0.0)	.05
Non-Hodgkin lymphoma	8	7.0 (7.0 to 7.1)	−1.9 (−2.1 to −1.8)	<.001	7.4 (7.3 to 7.4)	−1.9 (−2.1 to −1.7)	<.001	5.3 (5.1 to 5.4)	−1.7 (−2.2 to −1.2)	<.001	4.8 (4.6 to 5.1)	−1.4 (−1.9 to −0.9)	<.001	6.4 (5.4 to 7.5)	0.3 (−1.3 to 1.9)	.72	5.8 (5.6 to 6.0)	−1.4 (−1.7 to −1.0)	<.001
Esophagus	9	6.9 (6.8 to 7.0)	−1.9 (−2.9 to −0.9)	<.001	7.7 (7.7 to 7.8)	−1.5 (−2.6 to −0.3)	.01	5.2 (5.0 to 5.3)	−4.9 (−5.2 to −4.6)	<.001	2.7 (2.6 to 2.9)	−1.3 (−2.1 to −0.5)	.003	6.9 (6.0 to 8.0)	−1.1 (−2.7 to 0.6)	.20	3.7 (3.5 to 3.8)	−1.3 (−1.9 to −0.7)	<.001
Brain and other nervous system	10	5.4 (5.3 to 5.4)	0.4 (0.2 to 0.7)	.005	6.2 (6.1 to 6.3)	0.7 (0.4 to 0.9)	<.001	3.3 (3.1 to 3.4)	0.2 (−0.4 to 0.7)	.49	2.8 (2.6 to 2.9)	0.9 (−0.1 to 2.0)	.06	3.3 (2.7 to 4.0)	0.8 (−1.4 to 2.9)	.47	3.5 (3.4 to 3.7)	0.2 (−0.2 to 0.7)	.31
Kidney and renal pelvis	11	5.3 (5.3 to 5.4)	−2.4 (−3.3 to −1.4)	<.001	5.5 (5.4 to 5.6)	−0.9 (−1.1 to −0.7)	<.001	5.5 (5.3 to 5.7)	−1.0 (−1.4 to −0.7)	<.001	2.5 (2.4 to 2.7)	−2.8 (−4.8 to −0.8)	.01	9.8 (8.6 to 11.0)	−0.3 (−1.6 to 1.0)	.63	4.9 (4.8 to 5.1)	−0.9 (−1.4 to −0.4)	.002
Myeloma	12	4.1 (4.0 to 4.1)	−0.9 (−1.1 to −0.8)	<.001	3.9 (3.8 to 3.9)	−0.9 (−1.1 to −0.7)	<.001	7.5 (7.3 to 7.8)	−1.0 (−1.2 to −0.7)	<.001	2.0 (1.8 to 2.1)	−2.0 (−3.7 to −0.3)	.03	4.4 (3.6 to 5.3)	12.5 (−6.5 to 35.4)	.21	3.3 (3.2 to 3.5)	−0.5 (−1.2 to 0.1)	.11
Stomach	13	4.0 (3.9 to 4.0)	−2.5 (−2.7 to −2.3)	<.001	3.1 (3.0 to 3.1)	−2.8 (−3.2 to −2.5)	<.001	7.8 (7.6 to 8.0)	−3.1 (−3.4 to −2.9)	<.001	6.4 (6.2 to 6.7)	−3.7 (−4.2 to −3.2)	<.001	7.4 (6.4 to 8.5)	−3.1 (−4.7 to −1.4)	.001	6.3 (6.1 to 6.4)	−2.7 (−3.0 to −2.3)	<.001
Oral cavity and pharynx	14	3.9 (3.9 to 4.0)	0.6 (0.2 to 1.1)	.01	4.1 (4.0 to 4.1)	1.0 (0.6 to 1.4)	<.001	4.5 (4.4 to 4.7)	−2.8 (−3.4 to −2.2)	<.001	3.2 (3.0 to 3.4)	1.0 (−0.2 to 2.2)	.09	4.1 (3.4 to 4.9)	−0.4 (−2.5 to 1.8)	.73	2.4 (2.3 to 2.5)	−0.9 (−1.4 to −0.4)	.003
Skin melanoma	15	3.4 (3.3 to 3.4)	−5.7 (−6.9 to −4.5)	<.001	4.2 (4.2 to 4.3)	−5.9 (−7.4 to −4.5)	<.001	0.4 (0.4 to 0.4)	−1.5 (−3.2 to 0.2)	.08	0.4 (0.3 to 0.4)	–^f^	–	1.0 (0.7 to 1.5)	–^f^	–	0.9 (0.8 to 1.0)	−2.1 (−3.6 to −0.5)	.01
Non-melanoma skin	16	1.8 (1.7 to 1.8)	−0.1 (−2.5 to 2.4)	.94	2.0 (2.0 to 2.1)	0.4 (−1.8 to 2.7)	.70	0.7 (0.6 to 0.8)	−2.3 (−3.3 to −1.3)	<.001	0.4 (0.3 to 0.5)	–^f^	–	1.0 (0.7 to 1.5)	–^f^	–	0.8 (0.7 to 0.8)	0.4 (−0.9 to 1.6)	.54
Larynx	17	1.7 (1.7 to 1.7)	−2.5 (−2.6 to −2.3)	<.001	1.6 (1.6 to 1.7)	−2.1 (−2.3 to −1.9)	<.001	3.0 (2.9 to 3.2)	−3.6 (−4.0 to −3.2)	<.001	0.6 (0.5 to 0.6)	−3.3 (−5.2 to −1.5)	.002	1.9 (1.4 to 2.4)	–^f^	–	1.3 (1.2 to 1.4)	−4.7 (−6.3 to −3.0)	<.001
Soft tissue including heart	18	1.5 (1.5 to 1.6)	0.5 (0.3 to 0.7)	<.001	1.6 (1.6 to 1.6)	0.5 (0.2 to 0.8)	.001	1.6 (1.5 to 1.7)	0.6 (−0.2 to 1.4)	.13	1.0 (0.9 to 1.1)	0.8 (−0.8 to 2.4)	.32	1.5 (1.1 to 2.0)	–^f^	–	1.2 (1.1 to 1.3)	1.0 (0.1 to 1.9)	.03
Bones and joints	19	0.6 (0.6 to 0.6)	2.7 (0.6 to 4.8)	.01	0.6 (0.6 to 0.6)	0.5 (0.1 to 1.0)	.03	0.6 (0.6 to 0.7)	0.7 (−0.2 to 1.6)	.13	0.4 (0.3 to 0.4)	2.7 (0.7 to 4.7)	.01	0.7 (0.4 to 1.0)	–^f^	–	0.5 (0.4 to 0.5)	0.2 (−1.1 to 1.5)	.74
Females																			
Lung and bronchus	1	32.0 (31.9 to 32.1)	−4.3 (−4.8 to −3.7)	<.001	35.6 (35.4 to 35.7)	−3.9 (−4.4 to −3.4)	<.001	30.6 (30.2 to 30.9)	−4.5 (−5.3 to −3.7)	<.001	16.4 (16.0 to 16.8)	−4.6 (−6.6 to −2.6)	<.001	31.4 (29.7 to 33.3)	−2.3 (−3.1 to −1.5)	<.001	12.3 (12.1 to 12.5)	−4.2 (−6.2 to −2.1)	<.001
Breast	2	20.1 (20.0 to 20.2)	−1.0 (−1.4 to −0.7)	<.001	20.1 (20.0 to 20.2)	−0.9 (−1.2 to −0.6)	<.001	28.2 (27.9 to 28.5)	−1.4 (−1.6 to −1.3)	<.001	11.8 (11.5 to 12.1)	0.6 (−0.3 to 1.5)	.17	16.9 (15.6 to 18.3)	2.7 (−2.0 to 7.7)	.23	13.8 (13.6 to 14.1)	−1.1 (−1.3 to −0.9)	<.001
Colon and rectum	3	11.5 (11.4 to 11.6)	−2.0 (−2.4 to −1.6)	<.001	11.5 (11.4 to 11.6)	−1.7 (−2.1 to −1.3)	<.001	15.3 (15.0 to 15.5)	−3.1 (−3.3 to −2.9)	<.001	8.0 (7.8 to 8.3)	−2.1 (−2.6 to −1.6)	<.001	14.3 (13.1 to 15.5)	−0.8 (−2.0 to 0.4)	.17	8.6 (8.4 to 8.8)	−2.1 (−2.3 to −1.8)	<.001
Pancreas	4	9.6 (9.6 to 9.7)	0.2 (0.1 to 0.3)	<.001	9.6 (9.5 to 9.7)	0.1 (−0.1 to 0.3)	.18	12.3 (12.1 to 12.5)	−0.2 (−0.4 to −0.1)	.01	7.1 (6.9 to 7.4)	0.1 (−0.3 to 0.4)	.72	8.6 (7.7 to 9.6)	0.1 (−1.4 to 1.6)	.92	7.8 (7.7 to 8.0)	0.1 (−0.1 to 0.4)	.25
Ovary	5	6.7 (6.6 to 6.7)	−2.4 (−2.5 to −2.2)	<.001	7.1 (7.0 to 7.1)	−2.4 (−2.6 to −2.2)	<.001	6.0 (5.9 to 6.2)	−1.7 (−2.0 to −1.4)	<.001	4.4 (4.3 to 4.6)	−0.9 (−1.4 to −0.4)	.001	6.8 (6.1 to 7.7)	−1.4 (−2.7 to −0.2)	.03	5.1 (4.9 to 5.2)	−1.5 (−1.8 to −1.2)	<.001
Corpus and uterus, NOS	6	4.9 (4.9 to 5.0)	2.0 (1.6 to 2.3)	<.001	4.5 (4.5 to 4.6)	1.7 (1.4 to 2.1)	<.001	8.9 (8.8 to 9.1)	1.4 (0.5 to 2.3)	.006	3.3 (3.1 to 3.4)	2.5 (1.8 to 3.2)	<.001	4.0 (3.4 to 4.7)	–^f^	–	4.1 (3.9 to 4.2)	2.5 (1.9 to 3.1)	<.001
Leukemia	7	4.7 (4.7 to 4.7)	−1.4 (−1.6 to −1.2)	<.001	4.9 (4.9 to 5.0)	−1.3 (−1.5 to −1.2)	<.001	4.4 (4.3 to 4.5)	−1.5 (−1.8 to −1.2)	<.001	2.6 (2.5 to 2.8)	−0.3 (−6.3 to 6.0)	.92	3.3 (2.7 to 3.9)	−2.0 (−4.0 to 0.1)	.06	3.6 (3.5 to 3.8)	−0.8 (−1.2 to −0.4)	.001
Non-Hodgkin lymphoma	8	4.1 (4.1 to 4.2)	−2.8 (−2.9 to −2.7)	<.001	4.3 (4.3 to 4.4)	−2.8 (−3.0 to −2.7)	<.001	3.2 (3.1 to 3.3)	−2.2 (−2.5 to −1.8)	<.001	2.9 (2.8 to 3.1)	−2.0 (−2.6 to −1.5)	<.001	3.8 (3.1 to 4.4)	−3.0 (−4.7 to −1.2)	.002	3.7 (3.5 to 3.8)	−2.0 (−2.4 to −1.5)	<.001
Liver and intrahepatic bile duct	9	4.0 (4.0 to 4.1)	1.1 (0.5 to 1.6)	.004	3.6 (3.5 to 3.6)	2.1 (1.9 to 2.3)	<.001	4.9 (4.8 to 5.1)	1.7 (1.3 to 2.2)	<.001	5.5 (5.3 to 5.7)	−1.5 (−2.1 to −0.8)	<.001	7.9 (7.1 to 8.9)	0.9 (−0.9 to 2.7)	.31	6.0 (5.9 to 6.2)	1.2 (0.9 to 1.5)	<.001
Brain and other nervous system	10	3.6 (3.6 to 3.6)	0.4 (0.1 to 0.7)	.007	4.1 (4.0 to 4.1)	0.5 (0.2 to 0.8)	.003	2.3 (2.2 to 2.4)	0.6 (0.0 to 1.2)	.07	1.9 (1.8 to 2.1)	1.9 (0.9 to 2.9)	.001	2.8 (2.3 to 3.3)	–^f^	–	2.6 (2.5 to 2.7)	0.7 (0.3 to 1.2)	.003
Myeloma	11	2.6 (2.5 to 2.6)	−1.9 (−2.6 to −1.2)	<.001	2.3 (2.3 to 2.4)	−1.7 (−2.5 to −1.0)	<.001	5.2 (5.1 to 5.4)	−3.7 (−5.9 to −1.4)	.005	1.3 (1.2 to 1.4)	−0.9 (−2.2 to 0.4)	.16	3.4 (2.9 to 4.1)	−0.5 (−3.1 to 2.0)	.66	2.2 (2.1 to 2.3)	−1.3 (−1.8 to −0.7)	<.001
Kidney and renal pelvis	12	2.3 (2.2 to 2.3)	−1.4 (−1.6 to −1.3)	<.001	2.3 (2.3 to 2.4)	−1.4 (−1.6 to −1.2)	<.001	2.3 (2.2 to 2.4)	−1.7 (−2.1 to −1.2)	<.001	1.1 (1.0 to 1.2)	−1.2 (−2.1 to −0.2)	.03	3.7 (3.1 to 4.4)	−1.6 (−3.3 to 0.0)	.05	2.2 (2.1 to 2.3)	−0.7 (−1.2 to −0.2)	.01
Cervix uteri	13	2.2 (2.2 to 2.3)	−0.7 (−0.9 to −0.5)	<.001	2.0 (2.0 to 2.1)	−0.2 (−0.4 to 0.1)	.23	3.4 (3.3 to 3.5)	−2.4 (−2.7 to −2.1)	<.001	1.7 (1.6 to 1.8)	−2.2 (−3.1 to −1.2)	<.001	3.1 (2.5 to 3.6)	−1.8 (−3.5 to −0.1)	.04	2.6 (2.5 to 2.7)	−1.8 (−2.1 to −1.4)	<.001
Stomach	14	2.2 (2.1 to 2.2)	−1.9 (−2.2 to −1.5)	<.001	1.6 (1.5 to 1.6)	−3.0 (−3.2 to −2.8)	<.001	3.6 (3.5 to 3.7)	−3.4 (−3.6 to −3.1)	<.001	4.0 (3.9 to 4.2)	−3.2 (−3.7 to −2.6)	<.001	4.0 (3.3 to 4.7)	−2.4 (−4.1 to −0.7)	.009	3.9 (3.8 to 4.1)	−1.9 (−2.3 to −1.4)	<.001
Urinary bladder	15	2.1 (2.1 to 2.1)	−0.6 (−0.8 to −0.4)	<.001	2.2 (2.2 to 2.3)	−0.3 (−0.5 to −0.1)	.005	2.4 (2.3 to 2.5)	−1.5 (−1.9 to −1.1)	<.001	0.9 (0.8 to 1.0)	−1.1 (−2.1 to −0.2)	.02	1.9 (1.5 to 2.5)	–^f^	–	1.3 (1.2 to 1.4)	−0.6 (−1.4 to 0.1)	.08
Skin melanoma	16	1.4 (1.4 to 1.4)	−4.4 (−6.0 to −2.7)	<.001	1.8 (1.8 to 1.9)	−3.9 (−5.7 to −2.2)	<.001	0.3 (0.3 to 0.3)	−2.3 (−3.7 to −0.9)	.003	0.3 (0.3 to 0.4)	−0.4 (−3.0 to 2.1)	.72	0.5 (0.3 to 0.8)	–^f^	–	0.5 (0.5 to 0.5)	−1.6 (−2.7 to −0.5)	.008
Esophagus	17	1.4 (1.4 to 1.4)	−1.6 (−1.7 to −1.4)	<.001	1.5 (1.5 to 1.5)	−0.9 (−1.1 to −0.7)	<.001	1.6 (1.6 to 1.7)	−4.2 (−4.6 to −3.7)	<.001	0.7 (0.6 to 0.7)	−1.8 (−3.2 to −0.4)	.02	1.5 (1.1 to 1.9)	–^f^	–	0.7 (0.7 to 0.8)	−2.3 (−3.0 to −1.6)	<.001
Oral cavity and pharynx	18	1.3 (1.3 to 1.4)	−0.1 (−0.9 to 0.7)	.80	1.4 (1.4 to 1.5)	1.5 (−0.3 to 3.3)	.09	1.3 (1.2 to 1.3)	−2.1 (−2.7 to −1.5)	<.001	1.1 (1.0 to 1.2)	−1.7 (−2.8 to −0.5)	.01	1.3 (0.9 to 1.7)	–^f^	–	0.8 (0.7 to 0.8)	−0.4 (−1.4 to 0.5)	.35
Soft tissue including heart	19	1.2 (1.1 to 1.2)	0.1 (0.0 to 0.2)	.03	1.1 (1.1 to 1.2)	0.1 (−0.1 to 0.2)	.50	1.5 (1.4 to 1.6)	0.7 (0.3 to 1.1)	.001	0.8 (0.7 to 0.8)	0.4 (−1.0 to 1.9)	.52	1.0 (0.8 to 1.4)	–^f^	–	1.0 (0.9 to 1.0)	0.6 (−0.3 to 1.6)	.17
Gallbladder	20	0.7 (0.7 to 0.7)	−1.2 (−1.5 to −0.9)	<.001	0.6 (0.6 to 0.6)	−1.7 (−2.0 to −1.5)	<.001	1.0 (0.9 to 1.0)	−0.2 (−0.9 to 0.6)	.64	0.8 (0.7 to 0.8)	−0.8 (−1.9 to 0.3)	.14	1.7 (1.3 to 2.1)	−3.2 (−5.3 to −1.1)	.005	1.1 (1.0 to 1.2)	−1.7 (−2.5 to −0.8)	.001
Children (aged 0-14 y)																			
Brain and other nervous system	—	0.7 (0.7 to 0.7)	−0.3 (−0.7 to 0.1)	.13	0.7 (0.7 to 0.8)	−0.4 (−0.9 to 0.2)	.15	0.7 (0.7 to 0.8)	0.4 (−0.9 to 1.7)	.55	0.6 (0.5 to 0.7)	−0.8 (−2.8 to 1.3)	.42	1.0 (0.6 to 1.5)	–^f^	–	0.6 (0.6 to 0.7)	−0.2 (−1.0 to 0.6)	.67
Leukemia	—	0.5 (0.5 to 0.6)	−2.9 (−3.4 to −2.3)	<.001	0.5 (0.4 to 0.5)	−3.2 (−3.8 to −2.5)	<.001	0.5 (0.4 to 0.6)	−2.4 (−3.5 to −1.3)	<.001	0.5 (0.4 to 0.6)	−3.4 (−5.2 to −1.7)	.001	0.7 (0.4 to 1.1)	–^f^	–	0.7 (0.6 to 0.7)	−3.2 (−4.2 to −2.3)	<.001
AYA (aged 15-39 y)																–			
Female breast	—	2.2 (2.2 to 2.3)	0.5 (−0.6 to 1.6)	.35	2.0 (2.0 to 2.1)	0.6 (−1.0 to 2.3)	.44	4.0 (3.8 to 4.2)	−2.2 (−2.6 to −1.8)	<.001	1.4 (1.2 to 1.6)	−0.6 (−2.4 to 1.2)	.48	1.7 (1.1 to 2.6)	–^f^	–	1.8 (1.7 to 1.9)	−0.7 (−1.9 to 0.5)	.24
Brain and other nervous system	—	1.0 (0.9 to 1.0)	−0.1 (−0.6 to 0.3)	.52	1.2 (1.2 to 1.2)	0.0 (−0.5 to 0.5)	.95	0.7 (0.6 to 0.7)	0.5 (−0.6 to 1.5)	.35	0.6 (0.6 to 0.7)	1.3 (−0.3 to 3.0)	.11	0.9 (0.6 to 1.3)	–^f^	–	0.7 (0.6 to 0.7)	0.9 (0.0 to 1.8)	.06
Leukemia	—	0.9 (0.9 to 0.9)	−2.2 (−2.6 to −1.9)	<.001	0.8 (0.8 to 0.8)	−2.9 (−3.4 to −2.4)	<.001	1.0 (0.9 to 1.0)	−2.3 (−3.1 to −1.5)	<.001	0.8 (0.7 to 0.9)	−1.3 (−2.6 to 0.0)	.05	1.1 (0.8 to 1.5)	–^f^	–	1.2 (1.2 to 1.3)	−1.2 (−1.8 to −0.6)	<.001
Colon and rectum	—	0.9 (0.9 to 0.9)	0.9 (0.5 to 1.3)	<.001	0.9 (0.9 to 0.9)	1.1 (0.6 to 1.5)	<.001	1.2 (1.1 to 1.3)	0.6 (−0.1 to 1.3)	.11	0.7 (0.6 to 0.8)	−0.1 (−1.8 to 1.6)	.87	1.3 (0.9 to 1.8)	–^f^	–	0.7 (0.6 to 0.7)	1.7 (0.8 to 2.7)	.002

a Data are from the National Center for Health Statistics Public-Use data file for the total US, 1975-2018, covering the entire U.S. population (50 states and the District of Columbia). AAPC = average annual percent change; AI/AN = American Indian/Alaska Native; APC = annual percent change; API = Asian/Pacific Islander; CI = confidence interval; NOS = not otherwise specified; PRCDA = Indian Health Services Purchased/Referred Care Delivery Area.

b Cancers are sorted in descending order according to sex-specific rates for all racial/ethnic groups combined. More than 15 cancers may appear under males and females to include the top 15 cancers in every racial/ethnic group.

c Racial/ethnic groups are mutually exclusive. Data for non-Hispanic AI/AN persons are restricted to PRCDA counties.

d Rates are per 100 000 population and are age standardized to the 2000 U.S. standard population (19 age groups; Census publication p25 − 1130; U.S. Bureau of the Census, Current Population Reports, p25 − 1130. Washington, DC: U.S. Government Printing Office, 2000).

e AAPC is the average annual percent change and is a weighted average of the annual percent change (APCs) over the fixed interval 2014-2018 using the underlying Joinpoint model for the period of 2001-2018. Joinpoint models with up to 3 joinpoints are based on rates per 100 000 population age standardized to the 2000 U.S. standard population (19 age groups, Census P25–1130). Joinpoint Regression Program was used (version 4.8.0.1; Statistical Research and Applications Branch, National Cancer Institute; April 2020).

f The statistic could not be calculated. The average annual percent change was based on less than 10 cases for at least 1 year within the time interval.

**Table 4.** Joinpoint trends (2001-2018) for the most common causes of cancer death, all ages, all racial/ethnic groups combined by sex and age group, United States^a^

Sex and cancer site or type^b^	Trends in 2001-2018												2014-2018	
	1st segment			2nd segment			3rd segment			4th segment				
	Years	APC (95% CI)	*P*	Years	APC (95% CI)	*P*	Years	APC (95% CI)	*P*	Years	APC (95% CI)	*P*	AAPC^c^(95% CI)	*P*
All sites														
Both sexes combined	2001-2016	−1.5 (−1.5 to −1.5)	<.001	2016-2018	−2.3 (−3.1 to −1.6)	<.001	–	–	–	–	–	–	−1.9 (−2.3 to −1.6)	<.001
Males	2001-2015	−1.8 (−1.8 to −1.7)	<.001	2015-2018	−2.3 (−2.8 to −1.8)	<.001	–	–	–	–	–	–	−2.2 (−2.5 to −1.9)	<.001
Females	2001-2016	−1.4 (−1.4 to −1.3)	<.001	2016-2018	−2.1 (−2.9 to −1.2)	<.001	–	–	–	–	–	–	−1.7 (−2.1 to −1.4)	<.001
Children (aged 0-14 y)	2001-2018	−1.4 (−1.7 to −1.1)	<.001	–	–	–	–	–	–	–	–	–	−1.4 (−1.7 to −1.1)	<.001
AYA (aged 15-39 y)	2001-2005	−3.0 (−4.1 to −2.0)	<.001	2005-2018	−0.9 (−1.1 to −0.7)	<.001	–	–	–	–	–	–	−0.9 (−1.1 to −0.7)	<.001
Top 19 cancers for males														
Lung and bronchus	2001-2005	−2.0 (−2.3 to −1.7)	<.001	2005-2012	−2.9 (−3.1 to −2.8)	<.001	2012-2015	−4.0 (−5.0 to −3.0)	<.001	2015-2018	−5.7 (−6.2 to −5.1)	<.001	−5.2 (−5.6 to −4.9)	<.001
Prostate	2001-2013	−3.5 (−3.7 to −3.2)	<.001	2013-2018	−0.4 (−1.2 to 0.5)	.39	–	–	–	–	–	–	−0.4 (−1.2 to 0.5)	.39
Colon and rectum	2001-2005	−3.6 (−4.4 to −2.8)	<.001	2005-2012	−2.6 (−3.1 to −2.2)	<.001	2012-2018	−1.9 (−2.3 to −1.5)	<.001	–	–	–	−1.9 (−2.3 to −1.5)	<.001
Pancreas	2001-2018	0.3 (0.2 to 0.4)	<.001	–	–	–	–	–	–	–	–	–	0.3 (0.2 to 0.4)	<.001
Liver and intrahepatic bile duct	2001-2013	2.7 (2.5 to 2.9)	<.001	2013-2018	0.4 (−0.3 to 1.1)	.24	–	–	–	–	–	–	0.4 (−0.3 to 1.1)	.24
Leukemia	2001-2013	−1.0 (−1.1 to −0.8)	<.001	2013-2016	−3.1 (−5.1 to −1.0)	.007	2016-2018	−1.1 (−3.1 to 0.9)	.25	–	–	–	−2.1 (−3.4 to −0.8)	.001
Urinary bladder	2001-2013	0.1 (−0.1 to 0.3)	.53	2013-2018	−1.3 (−2.0 to −0.6)	.001	–	–	–	–	–	–	−1.3 (−2.0 to −0.6)	.001
Non-Hodgkin lymphoma	2001-2006	−3.1 (−3.7 to −2.4)	<.001	2006-2018	−1.9 (−2.1 to −1.8)	<.001	–	–	–	–	–	–	−1.9 (−2.1 to −1.8)	<.001
Esophagus	2001-2005	0.2 (−0.5 to 1.0)	.52	2005-2016	−1.1 (−1.3 to −0.9)	<.001	2016-2018	−2.7 (−4.9 to −0.5)	.02	–	–	–	−1.9 (−2.9 to −0.9)	<.001
Brain and other nervous system	2001-2006	−1.3 (−2.4 to −0.2)	.03	2006-2018	0.4 (0.2 to 0.7)	.005	–	–	–	–	–	–	0.4 (0.2 to 0.7)	.005
Kidney and renal pelvis	2001-2015	−0.8 (−0.9 to −0.6)	<.001	2015-2018	−2.9 (−4.3 to −1.5)	.001	–	–	–	–	–	–	−2.4 (−3.3 to −1.4)	<.001
Myeloma	2001-2018	−0.9 (−1.1 to −0.8)	<.001	–	–	–	–	–	–	–	–	–	−0.9 (−1.1 to −0.8)	<.001
Stomach	2001-2006	−3.7 (−4.5 to −2.9)	<.001	2006-2018	−2.5 (−2.7 to −2.3)	<.001	–	–	–	–	–	–	−2.5 (−2.7 to −2.3)	<.001
Oral cavity and pharynx	2001-2008	−1.5 (−2.4 to −0.7)	.002	2008-2018	0.6 (0.2 to 1.1)	.01	–	–	–	–	–	–	0.6 (0.2 to 1.1)	.01
Skin melanoma	2001-2009	0.9 (0.3 to 1.6)	.009	2009-2013	−0.9 (−3.6 to 1.9)	.48	2013-2018	−5.7 (−6.9 to −4.5)	<.001	–	–	–	−5.7 (−6.9 to −4.5)	<.001
Nonmelanoma skin	2001-2010	0.7 (−0.1 to 1.6)	.09	2010-2015	3.3 (0.8 to 6.0)	.02	2015-2018	−1.2 (−4.8 to 2.5)	.47	–	–	–	−0.1 (−2.5 to 2.4)	.94
Larynx	2001-2018	−2.5 (−2.6 to −2.3)	<.001	–	–	–	–	–	–	–	–	–	−2.5 (−2.6 to −2.3)	<.001
Soft tissue including heart	2001-2018	0.5 (0.3 to 0.7)	<.001	–	–	–	–	–	–	–	–	–	0.5 (0.3 to 0.7)	<.001
Bones and joints	2001-2013	−0.2 (−0.7 to 0.4)	.60	2013-2018	2.7 (0.6 to 4.8)	.01	–	–	–	–	–	–	2.7 (0.6 to 4.8)	.01
Top 20 cancers for females														
Lung and bronchus	2001-2007	−0.6 (−0.9 to −0.2)	.002	2007-2014	−2.0 (−2.3 to −1.7)	<.001	2014-2018	−4.3 (−4.8 to −3.7)	<.001	–	–	–	−4.3 (−4.8 to −3.7)	<.001
Breast	2001-2003	−1.5 (−2.6 to −0.5)	.01	2003-2007	−2.3 (−2.8 to −1.8)	<.001	2007-2014	−1.6 (−1.8 to −1.4)	<.001	2014-2018	−1.0 (−1.4 to −0.7)	<.001	−1.0 (−1.4 to −0.7)	<.001
Colon and rectum	2001-2010	−3.0 (−3.3 to −2.7)	<.001	2010-2018	−2.0 (−2.4 to −1.6)	<.001	–	–	–	–	–	–	−2.0 (−2.4 to −1.6)	<.001
Pancreas	2001-2018	0.2 (0.1 to 0.3)	<.001	–	–	–	–	–	–	–	–	–	0.2 (0.1 to 0.3)	<.001
Ovary	2001-2005	−1.0 (−2.0 to −0.1)	.04	2005-2018	−2.4 (−2.5 to −2.2)	<.001	–	–	–	–	–	–	−2.4 (−2.5 to −2.2)	<.001
Corpus and uterus, NOS	2001-2008	0.2 (−0.4 to 0.8)	.48	2008-2018	2.0 (1.6 to 2.3)	<.001	–	–	–	–	–	–	2.0 (1.6 to 2.3)	<.001
Leukemia	2001-2018	−1.4 (−1.6 to −1.2)	<.001	–	–	–	–	–	–	–	–	–	−1.4 (−1.6 to −1.2)	<.001
Non-Hodgkin lymphoma	2001-2018	−2.8 (−2.9 to −2.7)	<.001	–	–	–	–	–	–	–	–	–	−2.8 (−2.9 to −2.7)	<.001
Liver and intrahepatic bile duct	2001-2004	2.7 (1.0 to 4.5)	.007	2004-2008	0.6 (−1.0 to 2.2)	.41	2008-2013	3.4 (2.5 to 4.4)	<.001	2013-2018	1.1 (0.5 to 1.6)	.004	1.1 (0.5 to 1.6)	.004
Brain and other nervous system	2001-2006	−1.1 (−2.2 to 0.0)	.05	2006-2018	0.4 (0.1 to 0.7)	.007	–	–	–	–	–	–	0.4 (0.1 to 0.7)	.007
Myeloma	2001-2009	−2.5 (−3.0 to −2.0)	<.001	2009-2012	1.7 (−2.8 to 6.5)	.43	2012-2018	−1.9 (−2.6 to −1.2)	<.001	–	–	–	−1.9 (−2.6 to −1.2)	<.001
Kidney and renal pelvis	2001-2018	−1.4 (−1.6 to −1.3)	<.001	–	–	–	–	–	–	–	–	–	−1.4 (−1.6 to −1.3)	<.001
Cervix uteri	2001-2004	−2.9 (−5.1 to −0.7)	.01	2004-2018	−0.7 (−0.9 to −0.5)	<.001	–	–	–	–	–	–	−0.7 (−0.9 to −0.5)	<.001
Stomach	2001-2008	−3.0 (−3.5 to −2.4)	<.001	2008-2018	−1.9 (−2.2 to −1.5)	<.001	–	–	–	–	–	–	−1.9 (−2.2 to −1.5)	<.001
Urinary bladder	2001-2018	−0.6 (−0.8 to −0.4)	<.001	–	–	–	–	–	–	–	–	–	−0.6 (−0.8 to −0.4)	<.001
Skin melanoma	2001-2012	−0.3 (−1.0 to 0.4)	.34	2012-2018	−4.4 (−6.0 to −2.7)	<.001	–	–	–	–	–	–	−4.4 (−6.0 to −2.7)	<.001
Esophagus	2001-2018	−1.6 (−1.7 to −1.4)	<.001	–	–	–	–	–	–	–	–	–	−1.6 (−1.7 to −1.4)	<.001
Oral cavity and pharynx	2001-2009	−1.8 (−2.8 to −0.9)	.001	2009-2018	−0.1 (−0.9 to 0.7)	.80	–	–	–	–	–	–	−0.1 (−0.9 to 0.7)	.80
Soft tissue including heart	2001-2018	0.1 (0.0 to 0.2)	.03	–	–	–	–	–	–	–	–	–	0.1 (0.0 to 0.2)	.03
Gallbladder	2001-2018	−1.2 (−1.5 to −0.9)	<.001	–	–	–	–	–	–	–	–	–	−1.2 (−1.5 to −0.9)	<.001
Children (aged 0 − 14 y)														
Brain and other nervous system	2001-2018	−0.3 (−0.7 to 0.1)	.13	–	–	–	–	–	–	–	–	–	−0.3 (−0.7 to 0.1)	.13
Leukemia	2001-2018	−2.9 (−3.4 to −2.3)	<.001	–	–	–	–	–	–	–	–	–	−2.9 (−3.4 to −2.3)	<.001
AYA (aged 15 − 39 y)														
Female breast	2001-2010	−3.2 (−4.0 to −2.3)	<.001	2010-2018	0.5 (−0.6 to 1.6)	.35	–	–	–	–	–	–	0.5 (−0.6 to 1.6)	.35
Brain and other nervous system	2001-2018	−0.1 (−0.6 to 0.3)	.52	–	–	–	–	–	–	–	–	–	−0.1 (−0.6 to 0.3)	.52
Leukemia	2001-2018	−2.2 (−2.6 to −1.9)	<.001	–	–	–	–	–	–	–	–	–	−2.2 (−2.6 to −1.9)	<.001
Colon and rectum	2001-2018	0.9 (0.5 to 1.3)	<.001	–	–	–	–	–	–	–	–	–	0.9 (0.5 to 1.3)	<.001

^a^ Data are from the National Center for Health Statistics Public-Use data file for the total US, 1975-2018, covering the entire U.S. population (50 states and the District of Columbia). Joinpoint models with up to 3 joinpoints are based on rates per 100 000 persons are age standardized to the 2000 U.S. standard population (19 age groups, Census P25–1130). Joinpoint Regression Program was used (version 4.8.0.1; Statistical Research and Applications Branch, National Cancer Institute; April 2020). AAPC = average annual percent change; APC = annual percent change; CI = confidence interval; NOS, not otherwise specified.

^b^ Cancers are listed in descending rank order of sex-specific, age-adjusted death rates for 2014-2018 for all racial and ethnic groups combined. More than 15 cancers may appear under males and females to include the top 15 cancers in each racial and ethnic group.

^c^ AAPC is the average annual percent change and is a weighted average of the annual percent change (APC) over the fixed interval 2014-2018 using the underlying Joinpoint model for the period of 2001-2018.

During 2014-2018, overall cancer death rates decreased in every racial/ethnic group; the average annual pace of decrease ranged from 0.7% among AI/AN persons to 2.1% among API persons. The overall cancer death rate (per 100 000 population) was highest among Black persons (182.5), followed by rates among AI/AN (163.2), White (160.2), Hispanic (110.8), and API persons (98.0) (Figure 2). Death rates among males during 2014-2018 increased for 5 of the 19 most common cancers (bones and joints, oral cavity and pharynx, soft tissue including heart, brain and ONS, and pancreas); were stable for 3 cancers (liver, nonmelanoma skin, and prostate); and decreased for 11 cancers (Figure 3; Table 3). Death rates among females increased for 5 of the 20 most common cancers (uterus, liver, brain and ONS, pancreas, and soft tissue including heart), were stable only for oral cavity and pharynx cancer, and decreased for 14 cancers.

The 3 most common cancer deaths among males were lung, prostate, and colorectal cancer in most racial/ethnic groups except API males, in whom lung cancer was the most common cancer death, followed by cancer of the liver, colorectum, and prostate (Table 3). During 2014-2018, lung cancer death rates among males decreased in each racial/ethnic group. Prostate cancer death rates were stable among White, Black, and Hispanic males but decreased among API and AI/AN males. Colorectal cancer death rates were stable among AI/AN males but decreased in all other racial/ethnic groups. Pancreatic cancer was the fourth-most common cancer death among White and Black males, and death rates increased among White males but were stable in other racial/ethnic groups. Liver cancer was the fourth-most common cancer death among AI/AN and Hispanic males (and second among API males); death rates increased among White and AI/AN males, were stable among Black and Hispanic males, and decreased among API males.

Among females, the 3 most common cancer deaths were lung, breast, and colorectal in each racial/ethnic group, except among Hispanic females, in whom breast cancer was the most common and lung cancer was the second-most common (Table 3). Cancer death rates for these 3 cancers among females decreased during 2014-2018 in every racial/ethnic group, except for breast cancer death rates among API and AI/AN females and colorectal cancer death rates among AI/AN females, in whom rates were stable. Pancreas cancer was the fourth-most common cancer death among females in each racial/ethnic group. Although overall death rates of pancreas cancer slightly increased among females, there was no statistically significant increase in any racial/ethnic group. The largest increases in death rates among females during 2014-2018 were observed for uterine cancer (AAPC = 2.0%), with increasing rates among White, Black, API, and Hispanic females; the AAPC for AI/AN females could not be calculated.

In joinpoint analysis of death rates during 2001-2018, trends for several cancer types changed, notably for melanoma and cancers of the lung, kidney (males only), urinary bladder (males only), colorectum, female breast, prostate, and liver (Table 4). Some of the recent changes in death rates were favorable. Declines in lung cancer death rates accelerated; among males, for example, average declines in death rates per year were 2.0% during 2001-2005, 2.9% during 2005-2012, 4.0% during 2012-2015, and 5.7% during 2015-2018. Similarly, death rates of kidney cancer, which have declined since 2001 in both sexes, began declining more rapidly among males in 2015. Melanoma death rates started a considerable decline in 2013 among males (5.7% per year) and in 2012 among females (4.4% per year) after a period of stable trends. Similarly, bladder cancer death rates started to decline in 2013 among males (1.3% per year), although rates among females decreased steadily (0.6% per year) throughout 2001-2018. Furthermore, earlier increasing trends in liver cancer death rates stabilized among males and decelerated among females during 2013-2018. In contrast, earlier declines decelerated for colorectal and female breast cancers and stabilized for prostate cancer in recent years. For example, female breast cancer death rates decreased 2.3% per year on average during 2003-2007, 1.6% during 2007-2014, and 1.0% during 2014-2018. Prostate cancer death rates decreased 3.5% per year on average during 2001-2013 but then stabilized during 2013-2018.

Recent changes in death rates for several cancer types were consistent with changes in incidence rates, including decelerated declines in incidence rates for colorectal cancer and stabilized trends for liver cancer and prostate cancer (Table 2). Deceleration of declines in female breast cancer death rates coincided with a steady, slight increase in incidence rates during 2004-2017, which was preceded by a declining trend during 2001-2004. However, in contrast to declining death rates, melanoma incidence rates increased, and declines in lung cancer death rates outweighed declines in incidence rates.

### Stage-Specific Survival for Melanoma

**Figure 4.**
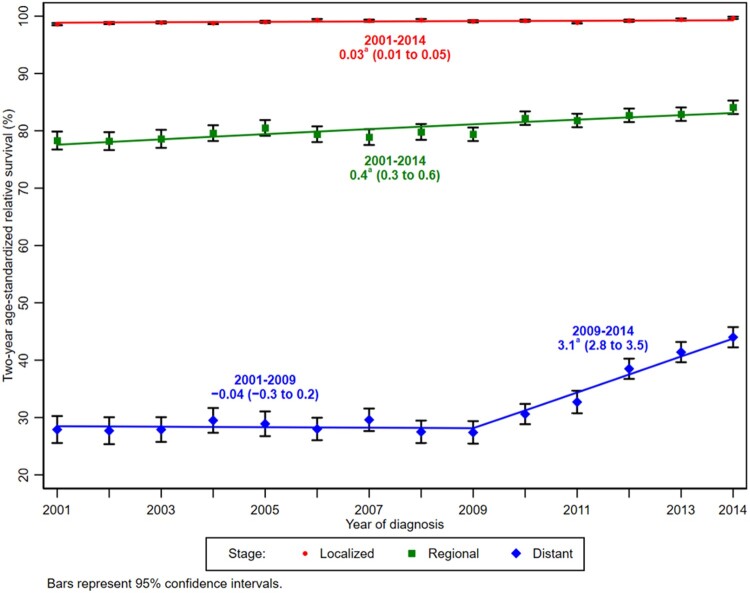
Trends in 2-year age-standardized relative survival for melanomas of the skin diagnosed during 2001-2014 and followed-up through 2016, both sexes and all races or ethnicities combined, by stage at diagnosis. Cases were censored at an achieved age of 100 years. Two-year relative survival estimates were age-standardized using the International Cancer Survival Standards, age standard 2, and age groups 15-44 years, 45-54 years, 55-64 years, 65-74 years, and 75 years and older. Trends were estimated using joinpoint regression (with up to 2 joinpoints) and characterized by the annual percent change (APC), the slope of a single segment. ^a^APCs were statistically significantly different from 0 based on proportional hazard joinpoint models fitted to survival data on the log hazard scale; 95% confidence limits are given in parentheses. Registries included for survival (28 states) covered 86% of the US population: Alabama, Arizona, California, Connecticut, Florida, Georgia, Illinois, Iowa, Kentucky, Louisiana, Maryland, Michigan, Minnesota, Missouri, New Jersey, New York, North Carolina, Ohio, Oklahoma, Oregon, Pennsylvania, South Carolina, Tennessee, Texas, Utah, Virginia, Washington, and Wisconsin.

The average 2-year age-standardized relative survival for melanoma cases diagnosed during 2010-2014 was 99.4% for localized-stage, 84.9% for regional-stage, and 41.6% for distant-stage tumors (Supplementary Table 1, available online). A notable change in trends was observed for distant-stage melanoma, for which stable 2-year age-standardized relative survival for cases diagnosed during 2001-2009 was followed by a 3.1% (95% CI = 2.8% to 3.5%) increase per year in 2-year relative survival for those diagnosed during 2009-2014 (Figure 4), with a similar pattern among males and females (Supplementary Table 1, available online). Two-year age-standardized relative survival increased 0.4% (95% CI = 0.3% to 0.6%) per year for regional-stage and 0.03% (95% CI = 0.01% to 0.05%) per year for localized-stage melanoma cases diagnosed during 2001-2014.

### Cancer Among Children

Among children aged 0-14 years, the incidence rate for all cancers combined was 16.8 cases per 100 000 standard population, ranging from 12.6 among AI/AN children to 17.8 among White children (Table 1). Overall cancer incidence rates increased during 2013-2017 (AAPC = 0.7%, 95% CI = 0.5% to 0.9%). The increase occurred in all racial/ethnic groups except among AI/AN children, in whom rates were stable (Table 1). The most common cancer types included leukemia (5.2 cases per 100 000 standard population), brain and ONS (3.8), and lymphoma (1.6), with increasing trends of 0.7% to 0.8% per year on average for each of these cancers during 2001-2017 (Table 2). Leukemia rates showed the most variability among racial/ethnic groups, ranging from 3.2 cases per 100 000 standard population among Black children to 6.2 among Hispanic children (Table 1). Leukemia incidence rates increased during the most recent 5 years (2013-2017) among White, Black, AI/AN, and Hispanic children but were stable among API children.

The cancer death rate among children was 2.1 deaths per 100 000 standard population, ranging from 1.8 among API children to 2.6 among AI/AN children (Table 3). The overall cancer death rate among children decreased during 2014-2018 (AAPC = −1.4%, 95% CI = −1.7% to −1.1%). The most common cancer deaths were from brain and ONS cancer (0.7 deaths per 100 000 standard population) and leukemia (0.5 per 100 000 standard population). During 2001-2017, death rates among children for brain and ONS cancer were stable and death rates from leukemia declined an average of 2.9% per year (Table 4).

### Cancer Among AYA

Among AYA aged 15-39 years, the overall cancer incidence rate was 75.9 cases per 100 000 standard population, ranging from 55.6 among API AYA to 84.4 among White AYA (Table 1). The most common cancer among AYA was female breast cancer (22.6 per 100 000 standard population), ranging from a rate of 17.7 among AI/AN AYA to a rate of 27.1 among Black AYA. The next most commonly diagnosed cancers were thyroid (12.1 per 100 000 standard population) and testicular cancer (11.0 per 100 000 standard population), with substantial variations in incidence rates by racial/ethnic group, being lowest among Black AYA (5.6 for thyroid and 2.6 for testis cancer) and highest among White AYA (13.8 for thyroid and 13.4 for testis cancer). Overall cancer incidence rates among AYA increased during 2001-2017 (APC = 0.9%, 95% CI = 0.8% to 1.0%), as did incidence rates of testicular cancer, whereas rates decreased for lymphoma and melanoma (Table 2). There were variations in trends during 2001-2017 for cancers of the colorectum, female breast, and thyroid. The annual percent increase in AYA colorectal cancer incidence rates almost tripled from 1.8% during 2001-2011 to 5.5% during 2011-2017. AYA female breast cancer incidence rates were stable during 2001-2010 then increased 1.1% per year during 2010-2017, whereas earlier increasing trends for AYA thyroid cancer stabilized during 2015-2017.

The cancer death rate among AYA was 8.9 deaths per 100 000 standard population and was highest among Black AYA (11.4 per 100 000 standard population) and AI/AN AYA (10.7 per 100 000 standard population) and lowest among API AYA (6.8 per 100 000 standard population) (Table 3). The most common cancer deaths among AYA were from female breast (2.2 deaths per 100 000 standard population), brain and ONS (1.0 per 100 000 standard population), leukemia (0.9 per 100 000 standard population), and colorectal cancer (0.9 per 100 000 standard population). Death rates from female breast cancer among Black AYA (4.0 per 100 000 standard population) were twice as high as among White AYA (2.0 per 100 000 standard population); rates were lower among API (1.4 per 100 000 standard population), AI/AN (1.7 per 100 000 standard population), and Hispanic AYA (1.8 per 100 000 standard population). Overall death rates among AYA declined during 2001-2018, with a faster average decline earlier (3.0% per year during 2001-2005) than in more recent years (0.9% per year during 2005-2018) (Table 4). Death rates for AYA female breast cancer declined 3.2% per year on average during 2001-2010 and then stabilized during 2010-2018. During 2001-2018, death rates increased 0.9% per year on average for AYA colorectal cancer, whereas rates decreased 2.2% per year for leukemia and were stable for brain and ONS cancer.

## Discussion

The decline in overall cancer death rates in the United States has accelerated in recent years, largely driven by accelerated declines in lung cancer death rates. Other favorable recent changes include a decline in melanoma death rates and stabilization (among males) and deceleration (among females) of earlier increasing trends in liver cancer death rates. In contrast, earlier declines in colorectal and female breast cancer death rates have slowed down, and declining trends for prostate cancer have stabilized in recent years. The observed trends in cancer death rates reflect changes in cancer risk factors (notably, cigarette smoking), screening, and treatment (17). In contrast to death rates, overall cancer incidence rates continue to increase among females and AYA and have stabilized among males after earlier declines, largely reflecting changes in cancer risk factors, notably increases in excess body weight (18). Changes in diagnostic practices have also influenced incidence trends for certain cancers, such as thyroid and prostate (19,20).

Death rates for multiple cancer types and overall remain higher among Black persons than in other racial/ethnic groups. The continuing disparity largely reflects a combination of multiple intertwined factors of tumor biology, stage at diagnosis, receipt of timely and effective treatment, and systemic discrimination in cancer care delivery (21,22). Furthermore, largely owing to social determinants of health inequalities, Black persons and individuals of lower socioeconomic groups in general are more likely to have a higher exposure to some cancer risk factors and limited access to healthy food, safe places for physical activity, and evidence-based cancer preventive services (17,23). Broad and multifaceted interventions can help close the racial mortality gap and address system failures across the continuum of care (24-26).

Historical declines in cigarette smoking have been followed by declines in incidence and death rates for several smoking-related cancers, including lung, bladder, and larynx. Moreover, as reported in previous studies (27,28) and observed in this study, lung cancer death rates have declined at a faster pace compared with declines in incidence rates since the mid-2000s, and the difference in the pace of decline has become greater in more recent years. This pattern corresponds to the timing of approval of targeted therapies and other advances in care for the most common subtype of lung cancer, non-small-cell lung cancer (NSCLC), which have resulted in increases in survival and accelerated declines in death rates for this subtype (28). In contrast, declines for small-cell lung cancer, with no approved novel treatments during our study period (29), have been similar to declines in incidence rates, with no improvement in survival rates for this subtype (28).

The first targeted therapy for NSCLC was approved by the FDA in 2003 (30), followed by the approval of several other targeted treatments, recommendations for genetic mutation testing of all individuals with nonsquamous NSCLC for relevant genetic targets in 2012 (28), and the approval of 3 immune checkpoint inhibitors for NSCLC in 2015-2016 (31). Increases in the proportion of individuals with NSCLC who receive definitive therapy (32), better understanding of treatment options for older persons with NSCLC (33–35), other recent advances in care for NSCLC, such as adjuvant therapy (36) and maintenance therapy (37), and increased access to care through the Medicaid expansion (38) may also have contributed to the acceleration in declines in lung cancer death rates. Any contribution of earlier detection through lung cancer screening (39) to declines in lung cancer death rates is likely to be modest, given low use of screening (4.4% of eligible individuals in 2015) (40). Despite substantial declines in death rates, lung cancer is still the leading cause of cancer death in both sexes, accounting for more than 22% of all cancer deaths in the United States (27), underscoring the need for broader implementation of tobacco control interventions.

In contrast to the rapid decline in incidence rates of smoking-related cancers, incidence rates continued to increase for several cancers, particularly those associated with excess body weight (41), such as cancers of the female breast, corpus uteri, pancreas, kidney, and myeloma (rates rising among females only). The staggering rise of obesity (42,43) and total sitting time (44) continues among both adults and youth. A parallel rise in death rates occurred for pancreatic and uterine cancers, and the earlier declines in death rates slowed for female breast and colorectal cancers, suggesting that for these cancers, increases in incidence rates are of sufficient magnitude to outweigh improvements in survival. Importantly, a recent study of trends by 5-year age groups showed steeper increases in incidence rates among progressively younger ages and successively younger generations for cancers of the corpus uteri, pancreas, kidney, colorectum, and multiple myeloma (18), foreboding of future burden of these cancers in the decades to come.

The declines in colorectal cancer incidence and death rates reflect changes in risk factors (such as reduced cigarette smoking), improvements in clinical management of the disease (affecting mortality only), and increases in colorectal cancer screening (45), which could reduce both mortality and incidence , because precancerous polyps can be detected and removed via colonoscopy (46). Declines in colorectal cancer incidence and death rates accelerated in the 2000s (47), largely owing to a substantial increase in the uptake of colorectal cancer screening among individuals aged 50-75 years, from 33.9% in 2000 to 58.7% in 2010, with colonoscopy use increasing from 19.1% in 2000 to 54.9% in 2010 (48). The deceleration of declines in colorectal cancer incidence and death rates in more recent years coincided with slower increases in uptake of screening since 2010, approaching 59.2% for colonoscopy and 62.4% for any screening test in 2015 (48). Moreover, in contrast to the overall declining trends, both incidence and death rates were rising in adults younger than 50 years, likely reflecting changes in risk factors such as obesity (18). The American Cancer Society lowered its recommended age for initiating colorectal cancer screening for average-risk individuals from 50 to 45 years of age in 2018 (46), and the US Preventive Services Task Force (USPSTF) released a draft statement in October 2020 that included a Grade B recommendation for screening individuals aged 45-49 years (49).

Female breast cancer is the most commonly diagnosed cancer among women and among AYA, and the second leading cause of cancer death among women. The increase of breast cancer incidence is largely driven by hormone-receptor positive cancer (50,51), which may in part reflect continuing reduction of parity rates (52,53), advanced age at first birth (53), the obesity epidemic (postmenopausal breast cancer) (52), high levels of physical inactivity, and increase of alcohol consumption (54,55). As noted in the last year’s report (56), the decline of breast cancer death rates overall has slowed since 2007, and even more so since 2014, and rates have stabilized among young women since 2010, halting the progress achieved through early detection and improved treatments. Moreover, substantial racial disparities persist, with death rates 40% higher among Black women than White women despite similar incidence rates.

Prostate cancer incidence rates stabilized from 2014 to 2017 after sharply declining during 2007-2014. These trends coincide with changes in use of prostate specific antigen (PSA) testing following the USPSTF recommendations against PSA-based screening for prostate cancer for men aged 75 years and older in 2008 (57) and for all men in 2012 (58). PSA testing for screening reasons sharply declined from 2007 to 2013 and remained unchanged from 2013 to 2015 (20,59). A recent analysis of prostate cancer incidence by stage at diagnosis, however, showed that rates continued to decline for local-stage disease, whereas rates increased substantially for regional and distant stage diseases (60). The increases in regional- and distant-stage diseases may have contributed to the recent stabilization of death rates after years of declining trends. Indeed, analysis of stage-specific prostate cancer death rates in the SEER areas showed that distant-stage prostate cancer death rates increased between 2012 and 2017, whereas rates for local-stage disease declined (61). Increases in prevalence of obesity (42), a suspected risk factor for fatal prostate cancer (41), may also have contributed to the recent stabilized mortality trend. Effects of the USPSTF recommendation for informed decision for PSA-based prostate cancer screening for men aged 55-69 years in 2018 (62) on prostate cancer incidence and death rates are yet to be determined.

Long-term increasing trends in uterine cancer death rates parallel trends in incidence, although death rates are increasing at a somewhat faster rate. Increasing uterine cancer incidence has been attributed to increasing obesity prevalence and decreased use of combined hormone replacement therapy (63,64). In our study, and as previously observed (63), incidence rates are similar for Black and White women, whereas uterine cancer death rates are twice as high in Black women. Similar to many other cancers, the disparity in death rates is associated with later stage at diagnosis and poorer quality of care (65) as well as higher frequency of nonendometrioid subtypes (which are more aggressive and have poorer prognosis) among Black women (66,67). Lack of correction for variation in hysterectomy rates can obscure overall trends and differences in incidence by racial/ethnic group, because the prevalence of hysterectomy in the United States has changed over time (eg, from 27.3% in 2000 to 23.9% in 2015) and varies by race/ethnicity (eg, 27.6% among Black women and 15.3% among Asian women in 2015) (67). A recent study, which analyzed hysterectomy-corrected uterine cancer incidence rates, found that rising rates are largely a result of the rapid increase in nonendometrioid subtypes among all racial/ethnic groups, which may in part explain slightly faster increases in uterine cancer death rates, although incidence rates of endometrioid subtypes, which account for about 75% of all uterine cancer cases, have also increased among Black, API, and Hispanic women (66). In addition to interventions to help women achieve and maintain a healthy body weight and physical activity level, promoting awareness of the importance of timely evaluation of abnormal vaginal bleeding has been shown to decrease the proportion of women diagnosed at more advanced stages (63).

Increasing trends in kidney cancer incidence rates in part reflect the obesity epidemic (18) and an increase in incidental detection of indolent or early-stage kidney cancers following a rise in the use of advanced imaging (68,69). Increases in the detection of indolent or early-stage cancer may also have contributed to declining trends in kidney cancer death rates, because prognosis for cancers confined to kidney is favorable, with a 5-year relative survival of 93% during 2009-2015 (27). Progress in treatment, such as targeted therapy for advanced kidney cancer, the first of which was approved by the FDA in 2005, may also have contributed to declining kidney cancer death rates (70). However, it is unclear why kidney cancer death rates declined in an accelerated rate during 2015-2018 among males. Similarly, bladder cancer death rates declined at an accelerated rate during 2013-2018 only among males. Declines in smoking, a main risk factor for bladder cancer (71), likely explain the slight, steady decline in bladder cancer death rates among females, because the declines parallels the incidence trends. The first new treatment for bladder cancer in almost 3 decades was approved by the FDA in 2016, likely with limited impact on survival in our study period (72).

As noted in the last year’s report (56), earlier increases in liver cancer death rates have decelerated among females and stabilized among males in more recent years. However, whereas incidence rates of liver cancer were increasing in last year’s report, rates have stabilized in both sexes in this year’s report. Because more than two-thirds of liver cancer cases are attributable to potentially modifiable risk factors (71), trends in liver cancer occurrence may largely reflect changes in risk factors. Historically, the birth cohort born during 1945-1965 has had substantially higher prevalence of hepatitis C virus infection, notably among men, and higher age-specific liver cancer death rates compared with other birth cohorts (73,74). Increases in liver cancer incidence and death rates in the past several decades coincided with the aging of the 1945-1965 birth cohort and a rise in obesity and type 2 diabetes (75). Several factors may have contributed to the stabilization or deceleration of increases in liver cancer incidence and death rates, including continued decline in liver cancer rates in API population (76), the introduction and dissemination of treatment for chronic hepatitis C virus infection over the past decade (77), and advances in liver cancer treatments (affecting mortality only) (78). However, use of these interventions and their impact varies across subpopulations, for example, by race/ethnicity or socioeconomic status (79,80), and the overall 5-year relative survival for liver cancer remains poor (33% for those diagnosed during 2009-2015) (27).

Last year’s report found that 5-year incidence trends for thyroid cancer had stabilized among both males and females after increasing for several decades. This year, for the first time, 5-year incidence rates are statistically significantly decreasing 2.0% per year among women of all racial/ethnic groups. Thyroid cancer incidence rates among AYA, which had been increasing, have now stabilized. However, incidence rates of advanced-stage thyroid cancer (81) and larger papillary thyroid cancers of classical variant (size ≥1 cm) have slightly increased in recent years (82), likely due to the obesity epidemic. As discussed in last year’s report, declines in overall thyroid cancer incidence are likely attributable to changes in diagnostic practices for low-risk tumors (19). A small proportion of the decline during 2015-2017 has been attributed to diagnostic coding changes for follicular variant of papillary thyroid carcinoma (82).

There are some other notable trends in this study. Reasons for declining incidence rates but increasing deaths rates for cancers of the brain and ONS are unclear and need further research. For cancer of the oral cavity and pharynx, incidence rates in both sexes and death rates among males are increasing. As shown in previous studies, the increasing incidence trends are limited to cancers in subsites with a strong association with human papillomavirus infection (such as tonsil and oropharynx), likely due to changes in sexual practices, whereas rates are declining in other subsites, largely due to declines in smoking prevalence (83). Similarly, incidence rates of anal and vulvar cancers—both associated with human papillomavirus—are increasing (84,85). In contrast, because of widespread dissemination of screening using the Papanicolaou test, the incidence rate of cervical cancer in the United States has been declining for several decades (85,86). However, studies by histological subtype have indicated that the decline has mainly been limited to cervical squamous cell carcinoma, the most common type of cervical cancer, whereas the incidence of cervical adenocarcinoma, which is less likely to be detected by Papanicolaou test compared with squamous lesions, has shown increasing trends among White women aged 30-59 years in recent years (87). Increases in incidence of childhood cancers overall and for major cancer types may be in part due to increases in exposure to risk factors, including obesity and radiation (88–90), although changes in classification of diseases or diagnostic procedures may also have contributed to increasing trends in some pediatric cancers (89).

As noted in the last 2 annual reports and in other studies, death rates for cutaneous melanoma have declined rapidly in recent years following introduction of new therapies, including targeted and immune checkpoint inhibitors, the first of which was approved by the FDA in early 2011 (15,56,91,92). In this year’s report, we present trends in stage-specific survival from 28 states and find increases in 2-year relative survival beginning in 2009 for distant-stage disease (3.1% per year). An increase in survival for distant-stage melanoma has also been documented in the CONCORD-3 study, in which 1-year net survival for cases diagnosed during 2001-2010 in the United States was stable at 43%, but it started to increase from 2010, reaching 56.6% for cases diagnosed in 2013 (93). The increase in late-stage melanoma survival in the United States slightly preceded the FDA approval of new therapies, likely because of the administration of these therapies through clinical trials and the FDA expanded access programs before the approval (93). A population-based study of treatment patterns and survival among patients in Ontario, Canada, documented increasing use of systemic therapy and new drugs from 2007 to 2015 (94). Two-year survival increased from 15% among those diagnosed in 2007-2009 to 35% for those diagnosed in 2014-2015; survival gains were most marked for patients treated with systemic therapy, which increasingly involved new drugs over the time period. Among these patients, 2-year survival increased from 16% in 2007-2009 to 44% in 2014-2015 (94).

The gains in survival for patients with metastatic melanoma are important indicators of potential progress in cancer treatment by introduction of new targeted and immune therapies. Recent research in melanoma treatment has focused on combination immunotherapies (95), optimal sequencing and combination of BRAF-MEK and immune checkpoint inhibitors, understanding the mechanisms of primary and acquired resistance to these therapies, and biomarkers for treatment selection and monitoring (96). Although advances in treatment of late-stage melanoma are promising, continuing efforts in primary prevention can reduce melanoma incidence rates, which are increasing among males and females. Recent analyses of incidence trends among non-Hispanic White persons show decreasing trends among younger adults, likely reflecting public health and policy interventions (97).

Results of this study represent national cancer trends, because mortality data cover the entire US population, incidence data cover 99% for incidence rates during 2013-2017 and 93% for incidence trends during 2001-2017, and melanoma survival data cover 86%. However, the study has several limitations. First, cancer rates for AI/AN, API, and Hispanic populations may be underestimated due to misclassification of data on race and ethnicity (98), and single-race population estimates derived from the original multiple-race categories may lead to some uncertainties about the population estimates (10). Moreover, data generally are available only for broad and heterogeneous racial/ethnic groups, whereas these groups may include subpopulations with very distinct cultural and health profiles. Second, although temporal trends for some cancer types may vary by histological or molecular subtype (50,51,99), we did not examine these patterns because they are beyond the scope of this report. Third, state cancer registries may have different methods of follow-up; however, these differences are unlikely to have substantially affected the findings in melanoma survival trends.

Cancer death rates in the United States continue to decline overall and for many cancer types. Furthermore, declines in death rates have accelerated for lung cancer and melanoma, likely owing to advances in treatment. However, earlier progress has slowed for several common cancers, death rates are increasing for several other cancers, and incidence rates are increasing slightly among females and in younger ages. These results inform ongoing and future efforts in prevention, early detection, and treatment and broad implementation of effective interventions, especially among under resourced populations.

## Funding

This work was supported by the American Cancer Society, the Centers for Disease Control and Prevention, the National Cancer Institute, and the North American Association of Central Cancer Registries. The American Cancer Society is a not-for-profit public health organization that receives support from the public through fundraising and direct contributions. The Society also receives a small portion of support from corporations and industry to support its mission programs and services.

## Notes

**Role of the funder:** The funding institutions had no role in the design and conduct of the study; collection, management, analysis, and interpretation of the data; preparation, review, or approval of the manuscript; and decision to submit the manuscript for publication.

##  

**Disclosures:** FI, AJ, JM, HS, KRY, and JZ are employed by the American Cancer Society, which receives grants from private and corporate foundations, including foundations associated with companies in the health sector for research outside the submitted work. The authors are not funded by any of these grants and their salary is solely funded through American Cancer Society funds, and they have nothing else to disclose. The other authors have no conflicts of interest to disclose.

##  

**Author contributions:** All authors: Conception and design. All authors: Data analysis and interpretation. FI, EMW, HS, and AJ: Drafted initial manuscript. All authors: Drafting, revising, editing manuscript.

##  

**Disclaimer:** The findings and conclusions in this article are those of the authors and do not necessarily represent the official positions of the authors’ agencies (the American Cancer Society, the Centers for Disease Control and Prevention, the National Cancer Institute, or the North American Association of Central Cancer Registries).

**Acknowledgements:** We gratefully acknowledge the contributions of the state and regional cancer registry staff and health department personnel for their work in collecting the data used in this report. In addition, we thank Information Management Services, Inc., for assistance in compiling the data used in this report.

## Data Availability

The data underlying this article cannot be shared publicly for analysis. The data belong to the state and territorial cancer registries and provided by the NAACCR for this purpose with permission. However, similar statistics can be generated using the CiNA on-line query systems: https://www.naaccr.org/interactive-data-on-line/. Additionally, approved researchers may request these data, which requires project-based approval by both NAACCR and each individual cancer registry. More information on data requests available here: https://www.naaccr.org/cina-data-products-overview/.

## Supplementary Material

djab131_Supplementary_Data
