# Supplementary material for: Annual Report to the Nation on the Status of Cancer, Part 1: National Cancer Statistics
Source: J Natl Cancer Inst. 2021 Jul 8;113(12):1648–69. doi: 10.1093/jnci/djab131 (PMC8634503; doi:10.1093/jnci/djab131)
Supplement: djab131_Supplementary_Data [file djab131_supplementary_data.pdf]

Supplementary Table 1. Trends in 2-year age-standardized relative survival for melanoma of the skin diagnosed during 2001–2014 and average 2-year age-standardized survival for melanomas diagnosed during 2011–2014 and followed up through 2016, by sex and stage at diagnosis, United States [a]

Stage at diagnosis	Trend 2001–2014				Average 2-year age-standardized relative survival for cases diagnosed during 2011–2014, % (95% CI)
	1st segment		2nd segment		
	Year of diagnosis	APC (95% CI)	Year of diagnosis	APC (95% CI)	
Overall					
Localized	2001–2014	0.03 (0.01 to 0.05) [b]			99.4 (99.3 to 99.5)
Regional	2001–2014	0.4 (0.3 to 0.6) [b]			84.9 (84.4 to 85.3)
Distant	2001–2009	−0.04 (−0.3 to 0.2)	2009–2014	3.1 (2.8 to 3.5) [b]	41.6 (40.6 to 42.5)
Unstaged	2001–2009	0.1 (−0.1 to 0.3)	2009–2014	0.7 (0.4 to 1.1) [b]	93.5 (93.2 to 93.9)
Male					
Localized	2001–2014	0.04 (0.01 to 0.07) [b]			99.2 (99.1 to 99.3)
Regional	2001–2007	0.1 (−0.3 to 0.4)	2007–2014	0.8 (0.6 to 1.1) [b]	83.8 (83.1 to 84.4)
Distant	2001–2009	−0.1 (−0.4 to 0.2)	2009–2014	3.0 (2.5 to 3.5) [b]	39.4 (38.2 to 40.6)
Unstaged	2001–2007	−0.2 (−0.5 to 0.2)	2007–2014	0.8 (0.5 to 1.1) [b]	92.7 (92.1 to 93.2)
Female					
Localized	2001–2014	0.03 (0.01 to 0.05) [b]			99.6 (99.5 to 99.7)
Regional	2001–2014	0.3 (0.2 to 0.4) [b]			86.6 (85.8 to 87.3)
Distant	2001–2009	0.03 (−0.3 to 0.4)	2009–2014	3.4 (2.9 to 3.9) [b]	45.3 (43.7 to 46.9)
Unstaged	2001–2014	0.3 (0.1 to 0.4) [b]			94.2 (93.7 to 94.7)

[a] Joinpoint models with up to two joinpoints. Cases were censored at an achieved age of 100 years. All 2-year relative survival estimates were age standardized using the International Cancer Survival Standards, age standard 2, and age groups 15-44, 45-54, 55-64, 65-74, and ≥75 years. Registries included for survival (28 states) covered 86% of the U.S. population: Alabama, Arizona, California, Connecticut, Florida, Georgia, Illinois, Iowa, Kentucky, Louisiana, Maryland, Michigan, Minnesota, Missouri, New Jersey, New York, North Carolina, Ohio, Oklahoma, Oregon, Pennsylvania, South Carolina, Tennessee, Texas, Utah, Virginia, Washington, Wisconsin. Data were from NPCR and SEER areas reported by NAACCR as meeting high−quality incidence data standards for the specified time periods. APC = annual percent change; CI = confidence interval; NAACCR = North American Association of Central Cancer Registries; NPCR = National Program of Cancer Registries; SEER = Surveillance, Epidemiology, and End Results.

[b] APC is statistically significantly different from zero.
